# Carbon Nanomaterials Interfacing with Neurons: An *In vivo* Perspective

**DOI:** 10.3389/fnins.2016.00250

**Published:** 2016-06-09

**Authors:** Michele Baldrighi, Massimo Trusel, Raffaella Tonini, Silvia Giordani

**Affiliations:** ^1^Nano Carbon Materials Laboratory, Istituto Italiano di TecnologiaGenova, Italy; ^2^Neuroscience and Brain Technology, Istituto Italiano di TecnologiaGenova, Italy

**Keywords:** carbon nanomaterials, *in vivo* studies, central nervous system, neuroprotection, drug delivery, imaging

## Abstract

Developing new tools that outperform current state of the art technologies for imaging, drug delivery or electrical sensing in neuronal tissues is one of the great challenges in neurosciences. Investigations into the potential use of carbon nanomaterials for such applications started about two decades ago. Since then, numerous *in vitro* studies have examined interactions between these nanomaterials and neurons, either by evaluating their compatibility, as vectors for drug delivery, or for their potential use in electric activity sensing and manipulation. The results obtained indicate that carbon nanomaterials may be suitable for medical therapies. However, a relatively small number of *in vivo* studies have been carried out to date. In order to facilitate the transformation of carbon nanomaterial into practical neurobiomedical applications, it is essential to identify and highlight in the existing literature the strengths and weakness that different carbon nanomaterials have displayed when probed *in vivo*. Unfortunately the current literature is sometimes sparse and confusing. To offer a clearer picture of the *in vivo* studies on carbon nanomaterials in the central nervous system, we provide a systematic and critical review. Hereby we identify properties and behavior of carbon nanomaterials *in vivo* inside the neural tissues, and we examine key achievements and potentially problematic toxicological issues.

## Introduction

In the last two decades carbon nanomaterials (CNMs) experienced an exponential increase in the number of application fields where they demonstrate excellent performances. As for many other nanomaterials, the interest from the scientific community on carbon nanomaterials is devoted to exploring their potential use in biomedicine in addition to engineering their application for goods manufacturing. Their nano-size enables to exploit unconventional interaction pathways with living systems (Freitas, 2005), for example allowing the delivery in the brain tissues of molecules that are usually rejected by the blood-brain barrier (BBB).

Carbon nanomaterials exhibit big diversity in structure, morphology, physical properties and chemical reactivity. Carbon nanotubes (CNTs), carbon nanohorns (CNHs), nanodiamonds (NDs), fullerenes, carbon nano-onions (CNOs), graphene and derivatives have emerged as promising classes of nanomaterials for imaging, diagnostic and therapeutic applications. Their atomic composition, i.e., carbon, has a much lower inherent toxic potential than the atomic species used in the manufacturing of other kinds of nanoparticles (usually transition metals or silica; Sohaebuddin et al., 2010; Sharifi et al., 2012). In addition, their peculiar physical properties and shapes display different interaction behaviors within cells and tissues, and their properties can be tailored by covalent and non-covalent functionalization that allows to modify their surface charge and to introduce fluorescent tags (Bartelmess et al., 2015a), cell-specific and disease-specific targeting molecules (Fabbro C. et al., 2012), Magnetic Resonance Imaging (MRI) contrast agents (Hahn et al., 2011), as well as drugs and nucleic acids (Bianco et al., 2005a; Cheung et al., 2010). Finally, the synthesis of raw carbon nanomaterials usually relies on very cheap sources and involves few synthetic steps, making cost-effective their large scale production (De Volder et al., 2013).

Carbon nanotubes are the most studied carbon nanomaterials for biomedical applications (Bianco et al., 2005b; Liu Z. et al., 2009; Gong et al., 2013; Lamberti et al., 2015). In the last few years, however, the scientific community has been showing a growing interest in graphene and graphene oxide (Zhang Y. et al., 2012; Zhang H. et al., 2013; Yang et al., 2013a), nanodiamonds (Mochalin et al., 2011; Perevedentseva et al., 2013) and carbon dots (Shen et al., 2012; Luo et al., 2013). On the opposite fullerenes, which attracted a lot of attention in the past, are now experiencing a gradual loss of interest due to concerns regarding toxicity (Zhu et al., 2006; Kolosnjaj et al., 2007; Partha and Conyers, 2009; Matija et al., 2013). Carbon nano-onions also are attracting attention for their possible biomedical application (Ghosh et al., 2011; Sonkar et al., 2012; Yang M. et al., 2013; Bartelmess et al., 2014, 2015b,c; Giordani et al., 2014; Frasconi et al., 2015a,b). Notably, it has been demonstrated either *in vitro* and *in vivo* that carbon nanomaterials can be efficiently degraded by means of enzymatic catalytic oxidation processes that are occurring either in plants, prokaryotes and eukaryotes (Kotchey et al., 2012, 2013; Bussy et al., 2015; Elgrabli et al., 2015; Sureshbabu et al., 2015) thus helping to dispel doubts regarding possible bioaccumulation hazards. A number of studies highlight high toxicity of carbon nanomaterials for fishes and amphibians (Zhu et al., 2006; Smith et al., 2007; Mouchet et al., 2008; Li J. et al., 2015). However, such non-specific and important toxicity is not observed in mammals, and studies regarding these species are not considered in this review.

Several efforts from the scientific community are devoted to investigate how carbon nanomaterials functionally interface with the central nervous system (CNS). There are great expectations on these materials since they show excellent compatibility with neuronal cells *in vitro* (Mattson et al., 2000; Webster et al., 2004; Li et al., 2011; Hopper et al., 2014), which makes them good candidates for the development of innovative diagnostic systems and therapeutic agents for brain pathologies such as neuronal or glial tumors. Moreover, the peculiar physical features of some of them, like the very high mechanical strength and the electrical conductivity, combined with their very low dimensions which provide an intimate contact with cells, enable a possible application both as support materials for neuroregeneration, e.g., after spinal cord injuries (Roman et al., 2011), as well as interface materials for high-efficiency recording and stimulation of the neuronal activity. Despite carbon nanomaterials are extensively probed for a number of biomedical applications using *in vivo* models (Yang K. et al., 2010; Gong et al., 2013; Perevedentseva et al., 2013; Hong et al., 2015), the number of studies dedicated to the CNS is substantially lower. It should be noted that in most cases the results are of great interest and undoubtedly depict a great potential for these materials.

In this review we focus our attention on the *in vivo* studies in the CNS in order to provide a comprehensive view of past and ongoing research in this field, highlighting the goals achieved, the interaction with neural tissues and the toxicity.

## Carbon nanotubes

**Figure 1:**
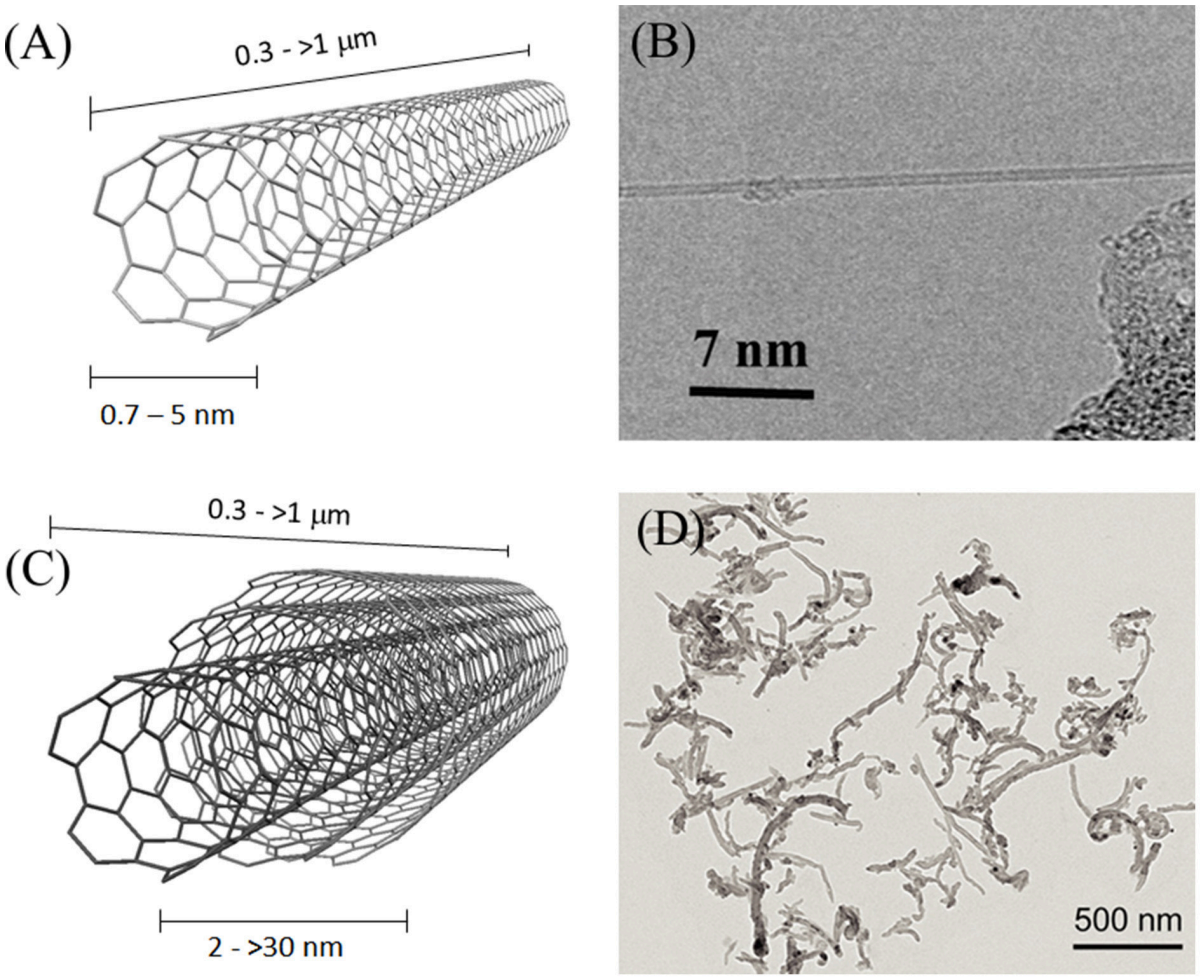
**Schematic representation of (A) SWCNTS and (C) MWCNTs. (B)** HRTEM micrograph of a single SWCNT; reprinted with permission from Zhang Y. et al. (2010), Copyright (2010) American Chemical Society. **(D)** HRTEM micrograph of MWCNTs, functionalized with N-methylpyrrolidine groups to improve their solubility in organic solvents; adapted from Cellot et al. (2011), copyright Society for Neurosciences (2011).

Carbon Nanotubes (CNTs) (Iijima, 1991) are the most-known and widest studied carbon nanomaterials. Their mechanical, thermal and electrical properties have been extensively investigated (Mintmire and White, 1995; Ruoff and Lorents, 1995; Salvetat et al., 1999; Odom et al., 2000; Dai, 2002; Cao et al., 2003; Popov, 2004), leading to their successful application in several commercial and prototype products (De Volder et al., 2013). From the structural point of view, CNTs consist of continuous rolled-up graphitic foils. They can be either single-walled (SWCNTs), if consisting of a single graphitic tube, or multi-walled (MWCNTs), if more concentric tubes are present. Their diameter ranges from 0.7 to 5 nm for SWCNTs and from 2 to >30 nm for MWCNTs, and their length can vary from a few hundreds of nm to several hundreds of microns (Figure 1). Although different fabrication methods are possible, chemical vapor deposition (CVD) using hydrocarbons as feed material and metal nanoparticles as catalyst is the most used (Cassell et al., 1999; Andrews et al., 2002). Several chemical reactions have been developed in order to modify their surface properties and to introduce functional molecules important for biological research (Tasis et al., 2006; Battigelli et al., 2013a).

The use of CNTs for the development of new diagnostic and therapeutic agents is of primary interest in biomedical research. Carbon nanotubes are successfully applied in sensing, imaging, drug delivery, and also nucleic acid delivery applications both in single cells and *in vivo* (Kateb et al., 2007; Ladeira et al., 2010; Wu et al., 2010; Al-Jamal et al., 2011; Liu Z. et al., 2011; Bates and Kostarelos, 2013; Battigelli et al., 2013a,b; Hong et al., 2015).

Toxicity of carbon nanotubes is a matter of debate: a number of studies highlight toxic effects in cells upon CNTs exposure (Muller et al., 2005; Magrez et al., 2006; Smith et al., 2007; Mouchet et al., 2008; Sharifi et al., 2012; Li J. et al., 2015). It should be noted, however, that a great contribution to these adverse effects could be led back to Fe, Ni, Co, and Y nanoparticles deriving from the CNTs synthesis, that are still present in variable amounts in raw CNTs samples. The careful removal of metal contaminants as well as chemical functionalization in fact lead to a drastic reduction of the nanomaterial toxicity (Pulskamp et al., 2007; Movia et al., 2011; Movia and Giordani, 2012). A further source of concerns is their similar behavior to that of asbestos fibers: their tendency to aggregate in bundles (especially for unfunctionalized CNTs) can lead to the occurrence of important inflammatory responses (Poland et al., 2008), which can be alleviated by improving the nanomaterial dispersibility thanks to its covalent or noncovalent functionalization with polar moieties (Ali-Boucetta et al., 2013). In experiments involving neuronal cells, which are commonly considered particularly sensitive to toxicants and inflammation, high purity and functionalized carbon nanotubes seldom show toxicity (Bardi et al., 2009; Gaillard et al., 2009; Vittorio et al., 2009; Yang Z. et al., 2010; Zhang Y. et al., 2010, 2011; Bussy et al., 2015). Finally, it has been discovered that CNTs can be enzymatically degraded by peroxidases (Kotchey et al., 2012, 2013) in macrophages (Kagan et al., 2014), eosinophils (Andón et al., 2013), neutrophyls (Bhattacharya et al., 2014), and microglia (Bussy et al., 2016), as well as in the extracellular space (Farrera et al., 2014), thus mitigating the concerns regarding possible toxic effects due to their accumulation inside the body.

CNTs are permissive substrates for the adhesion and growth of primary neurons (Mattson et al., 2000; Hu et al., 2004; Gabay et al., 2005; Gheith et al., 2005; Lovat et al., 2005; Dubin et al., 2008; Gaillard et al., 2009; Kam et al., 2009; Tran et al., 2009; Jin et al., 2011; Park et al., 2011a). They also promote stem cells differentiation into neurons (Chao et al., 2009; Park et al., 2011a), action potential appearance in immature neurons (Fabbro et al., 2013) and they stimulate the propagation of dendritic backcurrents in isolated neurons (Cellot et al., 2009). Collectively, this evidence suggests their possible use for the therapy of neurodegenerative pathologies and spinal cord injuries. Moreover, CNTs are applied to record and stimulate neural activity in single neurons, artificial ganglia and spinal cord sections (Gheith et al., 2006; Mazzatenta et al., 2007; Kam et al., 2009; Shein et al., 2009; Shoval, 2009; Cellot et al., 2011; Fabbro A. et al., 2012; David-Pur et al., 2014). Microelectrodes coated with CNTs show enhanced sensitivity in neuronal activity recordings compared to state of the art devices (Keefer et al., 2008; Jan et al., 2009; Luo et al., 2011). Finally, CNTs are also able to deliver functional molecules inside neurons (Kateb et al., 2007; Wang C.-H. et al., 2009; Cellot et al., 2010; Ren et al., 2012).

CNTs show in general good compatibility *in vivo* with neuronal tissues. Intravenous (*i.v*.) administration of ^13^C-enriched SWCNTs in mice (Yang et al., 2007) demonstrates that these nanomaterials (10–30 nm × 2–3 μm bundles) are able to cross the BBB and accumulate inside the brain tissues, although to a little extent. Furthermore, this study indicates that SWCNTs do not show acute toxicity despite their accumulation in several organs (especially liver, lungs, and spleen) and their low clearance. However, it has to be underlined that the long persistency of SWCNTs in lungs and in the liver can provide moderate toxicity in these organs (Yang et al., 2008).

A very recent report indicates that high doses of PEG-SWCNTs (1–10 μm bundles) display toxicity when stereotactically injected in rat hippocampus (Dal Bosco et al., 2015). Apparently PEG-SWCNTs are impairing contextual fear memory after long-term exposure at 0.5 and 1 mg/mL concentrations because of the oxidative stress generated by the nanomaterial. Although this study highlights how an eventual accumulation of CNTs inside the brain tissues can potentially lead to toxic effects, it is extremely unlikely that such high concentrations can be reached in localized regions of the CNS, unless local administration is used. Moreover, no explanation can be found to the evidence that higher concentrations of PEG-SWCNTs (2.1 mg/mL) do not cause oxidative stress and in general toxicity in the hippocampal tissues.

**Figure 2:**
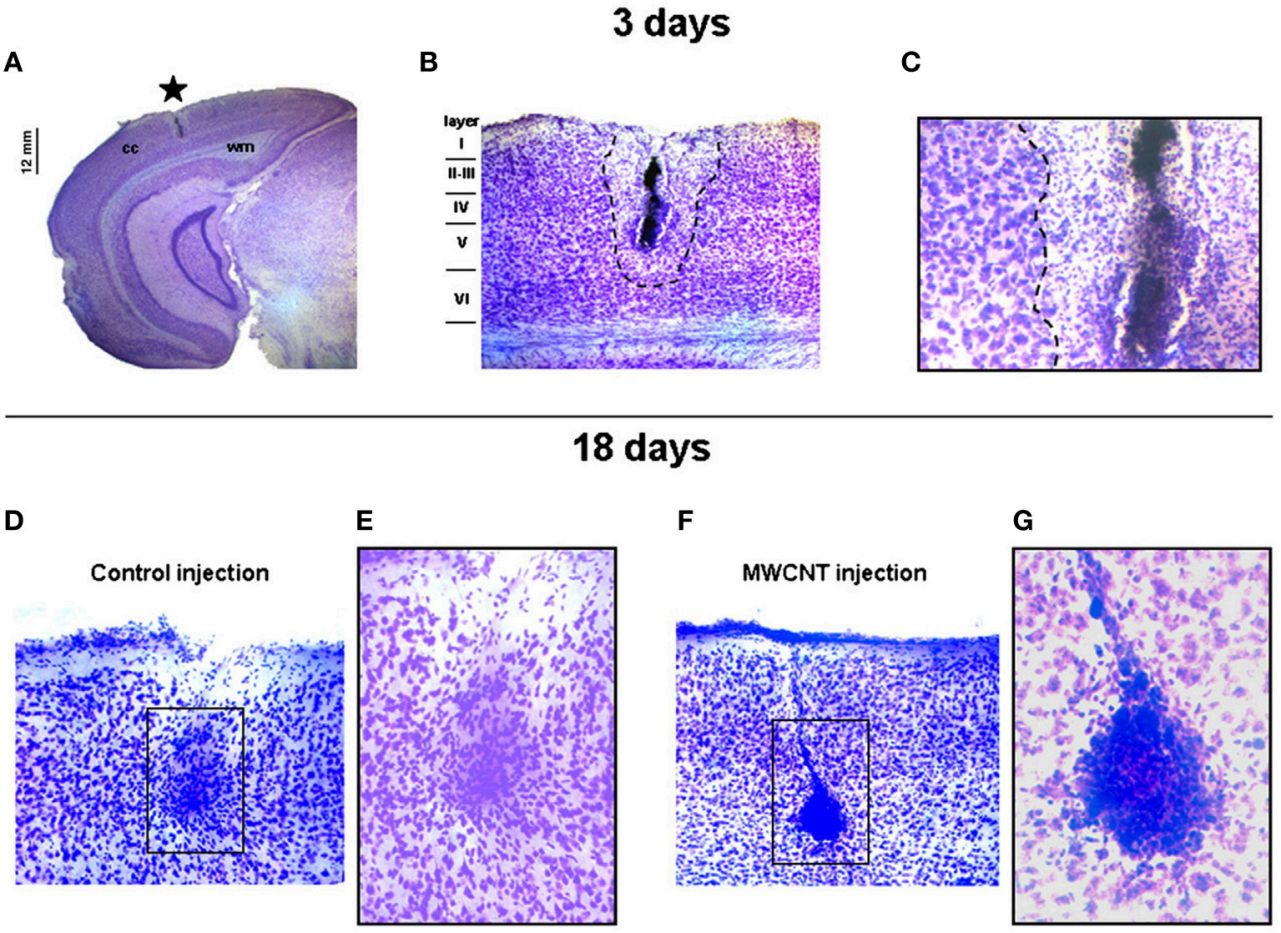
**Coronal brain slices showing short and long term effects of PF-127 coated MWCNTs intracortical injection. (A)** Localization of the injection site (star); cc, cerebral cortex; wm, white matter. **(B,C)** Magnifications of the injection site 3 days after the injection: outside the lesion area (dashed line) cerebral tissues show normal neuronal density and tissue layering. **(D,E)** Control mice and **(F,G)** PF-127 coated MWCNTs injected mice brain slices 18 days after the injection: both the lesion sites present normal gliosis surrounding the injection site. Reprinted from Bardi et al. (2009), Copyright (2009), with permission from Elsevier.

MWCNTs display in general a high biocompatibility *in vivo* with neural tissues: direct injection of a suspension of MWCNTs (10–30 nm × 2 μm) coated with the nonionic surfactant Pluronic F127 (PF-127) in mice visual cortex (Bardi et al., 2009) results in no substantial morphological differences observed in brain tissues comparing to a control injection, also in terms of injection lesion volume. Later timepoint analysis too revealed no sign of damage to the surrounding tissues apart from the expected gliosis engulfing the nanotubes (Figure 2). MWCNTs are also able to cross the BBB: [^111^In]-DTPA-MWCNTs (20 nm × 0.5 μm) administered *in vivo* by tail vein injection (Kafa et al., 2015) display a maximum brain accumulation of 1.1% of injected dose per gram of tissue 5 min after the injection, followed by a gradual slow excretion. Micropinocytosis from the perivascular epithelial cells seems in this case to be the main internalization mechanism and therefore transcellular uptake is hypothesized as the primary mechanism for the BBB crossing (Kafa et al., 2015). As SWCNTs however, also MWCNTs tend to accumulate in the liver and the lungs where they can possibly produce toxicity in the long term.

Their morphological characteristics and their tendency to agglomerate are found to play a role in determining the nanomaterial's fate and inflammatory potential inside the brain: long ammonium MWCNTs (MWCNTs-${\text{NH}}_{3}^{+}$, 20–30 nm × 0.5–1 μm) and short oxidized ammonium-MWCNTs (ox-MWCNTs-${\text{NH}}_{3}^{+}$, 20–30 nm × 0.2–0.3 μm) display in fact remarkable differences after direct local injection in mice motor cortex (Bardi et al., 2013). Short ox-MWCNTs-${\text{NH}}_{3}^{+}$ are confined in a very narrow area forming compact agglomerates and they can be found into the cytoplasm exclusively inside vesicles. Moreover, they show inflammatory potential, although it should be underlined that within 1 week the expression levels of the inflammatory cytokines return to normality. On the contrary, long MWCNTs-${\text{NH}}_{3}^{+}$ distribute over a very large area, they are found into the cells both inside vesicles and free-floating in the cytoplasm and have low inflammatory potential. Remarkably, microglia is found to be able to degrade long MWCNTs-${\text{NH}}_{3}^{+}$ even at early time points (Nunes et al., 2012), providing partial to complete loss of their morphology. Also, it has been recently demonstrated that different CNTs functionalizations can vary the short-term kinetics of CNTs biodegradation by microglia (Bussy et al., 2016), however not providing relevant differences in the nanomaterial's long-term fate. These evidences indicate that the possible accumulation of CNTs, which may eventually produce toxic effects, can be efficiently prevented also in the brain thanks to the natural body defense mechanisms.

**Figure 3:**
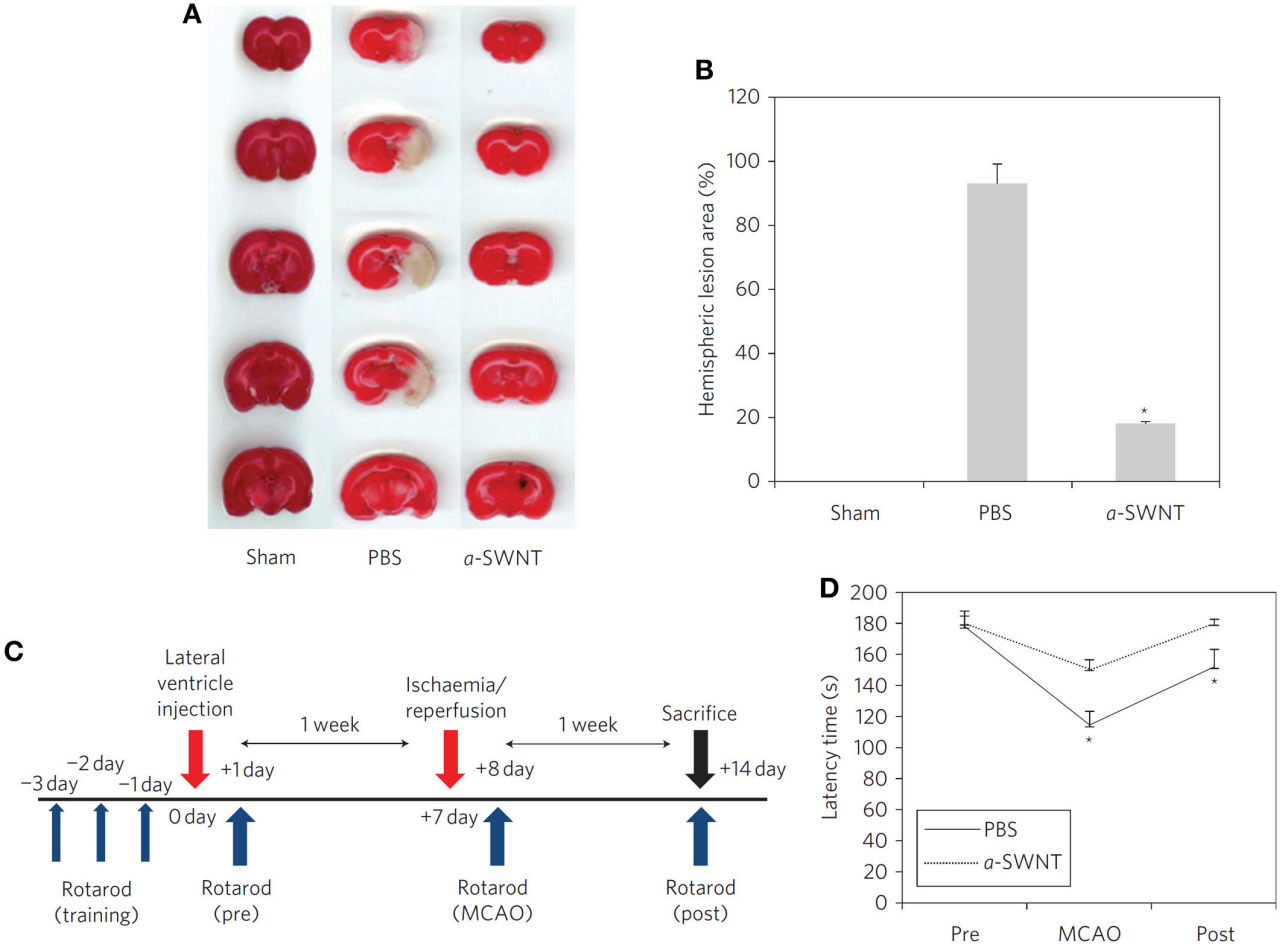
**Morphological and functional neuroprotective effects of the SWCNTs-NH_2_pretreatment after ischemia-reperfusion. (A)** Coronal brain sections (stained with tetrazolium chloride) of sham, PBS and SWCNTs-NH_2_ (here called a-SWNT) treated mice, where white areas correspond to the infarcted regions after MCAO. **(B)** Quantification of the lesion in the brain sections showed in **(A)**. **(C)** Schedule of motor functionality experiments. **(D)** Motor coordination results from Rotarod tests indicating complete recovery of motor coordination in SWCNTs-NH_2_ treated mice. Data reported as mean + s.e.m. ^*^P < 0.001 vs. pre-MCAO. Reprinted by permission from Mcmillan Publishers Ltd.: Nature Nanotechnology, Lee H. J. et al. (2011), Copyright (2011).

Besides the compatibility studies, CNTs have been also probed *in vivo* for their possible use as therapeutic agents, in particular as neuroprotectants against ischemic damages. In this context, CNTs covalent amino/ammonium derivatives and their further modifications display very promising results. SWCNTs amino derivatives (SWCNTs-NH_2_, 4–10 nm × 0.5–1.5 μm) are able to drastically reduce the brain damages induced by stroke when preventively administered in lateral ventricles (Lee H. J. et al., 2011): after surgical transitory middle cerebral arteria occlusion (MCAO), SWCNTs-NH_2_ treated rats display a much lower cerebral infarction volume with respect to untreated rats. Also apoptosis, inflammatory, neurogenesis and angiogenesis levels in SWCNTs-NH_2_ treated rats' brains indicate that the nanotubes are effective in reducing cell death and inflammatory response and in promoting neuroregeneration. Most impressively, a complete restoring of motor function can be achieved in rats 7 days after the ischemic insult (Figure 3). The therapeutic efficacy of CNTs against brain ischemic damages can be improved by using the nanomaterial in order to deliver neuroprotective siRNAs. Ammonium-MWCNTs (MWCNTs-${\text{NH}}_{3}^{+}$, 20–30 nm × 0.5–2 μm), are known in fact to be efficient siRNA delivery systems (Al-Jamal et al., 2010), and can be loaded with Caspase-3 siRNA (*siCAS3*), which is able to inhibit the expression of caspase-3, an enzyme involved in apoptosis (Al-Jamal et al., 2011). Preventive administration of the nanomaterial inside rats brain parenchyma and its internalization by neurons within 48 h from the injection can therefore guarantee motor ability retention in rats after the induction of the ischemic insult. The combined neuroprotective effect of the nanomaterial and of the siRNA is particularly evident if considering that, after the ischemic insult, treated animals brains show apoptosis markers levels that are substantially similar to those of healthy animals. Unfortunately, similar results could not be obtained if the nanomaterial is administered after the stroke event.

Drug delivery into the brain is also one of the most desired biomedical applications of nanomaterials. By simply exploiting that unfunctionalized—but shortened—SWCNTs (0.8–1.2 nm × 50–300 μm) administered through the gastrointestinal (GI) tract are able to cross the BBB and preferentially localize in neurons' lysosomes, it is possible to use these same SWCNTs to deliver Acetylcholine (ACh) in Alzheimer's disease (AD) model mice's brains through the GI tract. Once in the neuronal lysosomes, the acidic pH triggers the release of the drug from the ACh-SWCNTs to the neuron cytosol, providing the recovery of the mice's learning abilities (Yang Z. et al., 2010). Also the more bulky MWCNTs can show very interesting drug delivery abilities in the CNS, when opportunely functionalized in order to improve their dispersibility in aqueous media; moreover, the concomitant grafting of targeting biomolecules to the nanomaterial scaffold increases the specificity of the therapeutic action: doxorubicin (DOX) loaded oxidized MWCNTs (DOX-oMWCNTs, 10 nm × 5–15 μm) possessing a PEG unit and grafted with angiopep-2 (ANG, a peptide targeting both the BBB and the LRP receptor expressed by glioma cells) demonstrate to be highly effective against glioma (Ren et al., 2012). Angiopep-2-targeted oMWCNTs are able to cross the BBB in higher quantity with respect to unfunctionalized or PEG-DOX functionalized MWCNTs, and to accumulate more selectively into the tumor mass. As result, mice treated with DOX-oMWCNTs-PEG-ANG show a 20% increase in survival compared to mice treated with the untargeted nanomaterial and by 42% with respect to mice treated with only DOX. DOX-oMWCNTs-PEG-ANG also display a little higher liver and spleen accumulation than DOX and DOX-oMWCNTs-PEG but lower kidney and lung accumulation, and a markedly reduced cardiac toxicity with respect to DOX, a characteristic which also represents a remarkable improvement.

Efficient delivery of therapeutic genetic material in the CNS can be also achieved thanks to SWCNTs. Alongside with the previously mentioned delivery of siRNA for ischemic damage reduction purposes, SWCNTs, and in particular PEG-functionalized SWCNTs (1–3 nm × 0.2–0.4 μm), are able to deliver *CpG* oligonucleotide (*CpG*-CNTs), which has antitumor activity via activation of TLR9-mediated immune response, to the tumor-associated inflammatory cells in brain implanted glioma in mice (Zhao et al., 2011). The intracranial injection of *CpG*-CNTs provides the recruitment of Natural Killer (NK) and CD8^+^ cells and the development of immune response against the glioma cells, which results in tumor cells depletion and survival of 50–60% of treated mice, while no survival is observed when mice are treated with a single dose of non-conjugated *CpG* oligonucleotide. Moreover, the adverse effects commonly associated with the standard *CpG* antitumor therapy are not observed when using *CpG*-CNTs. Finally, the surviving treated mice develop immunity against glioma, therefore they undergo spontaneous remission of the tumor when this is re-injected into their brains.

CNTs were also probed for neuroregeneration applications in spinal cord injury (SCI) model rats. Post-injury administration of PEG-functionalized SWCNTs (PEG-SWCNTs) in the lesion site is found to promote axonal survival and repair, while delayed administration is able to achieve a dose-dependent reduction in the lesion volume in both gray and white matter, and an increase in the number of neuronal fibers in the lesion epicenter with a modest sprouting of corticospinal tract axons into this region (Roman et al., 2011). Neither alterations in reactive astrogliosis at the lesion site nor toxicity or neuropathic pain are present. As outcome, a dose-dependent moderate recovery of motility in treated rats is achieved.

Taking into account the studies above mentioned, carbon nanotubes emerge as extremely versatile materials for a number of useful applications in the CNS. Besides their single-wall or multi-wall nature, appropriately functionalized CNTs demonstrate to be good therapeutic agents against ischemic damage as well as excellent vectors for drug delivery in the CNS. Apparently MWCNTs are preferred to SWCNTs for delivery applications in the CNS, despite the fact that the latter display a higher specific surface area and therefore a higher loading capacity. Economical reasons may also play a role in the choice. CNTs show high biocompatibility with the brain tissues, contrary to the data reported in some cells studies and when administered in the lungs and in the GI tract. Important in this sense is that the CNTs used in these studies have been in general functionalized with highly polar moieties or they display structural characteristics that prevent their excessive aggregation in aqueous media, which can potentially give rise to immune response. Furthermore CNTs also demonstrate the ability to mitigate the toxicity of some drugs. In summary, we believe that CNTs have to be still considered cutting-edge nanomaterials for the therapy of CNS diseases.

## Fullerenes

**Figure 4:**
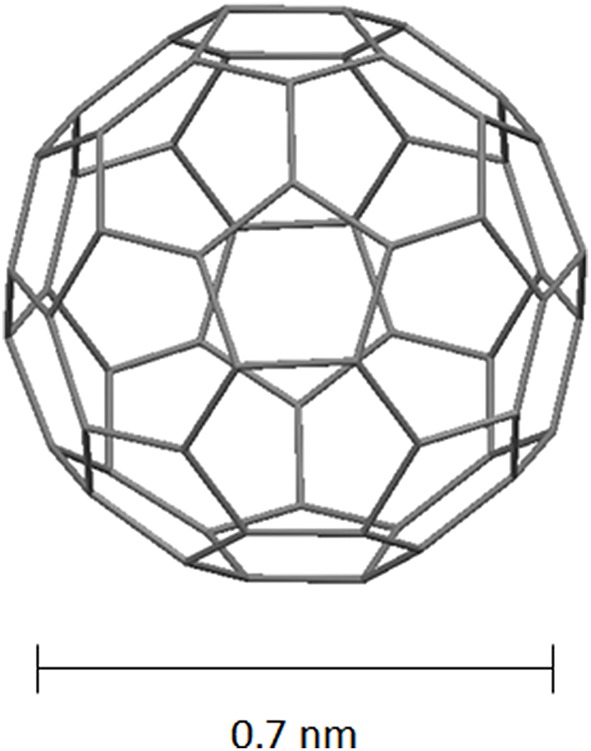
**Schematic representation of C_60_ fullerene**.

Fullerenes are defined molecular entities with a precise atomic composition and hollow spherical shape. Buckminsterfullerene, better known as C_60_ fullerene, is the first and the smallest stable fullerene isolated, and the most studied because of its relative ease of synthesis. It is obtained in relatively good yields from graphite using the arc-discharge technique, and purified from byproducts by solvent extraction followed by chromatography. In its structure the 60 sp^2^-hybridized carbon atoms arrange to form a truncated icosahedron structure with a diameter of 0.7 nm (Figure 4). Several derivatives with hydrophilic (carboxyfullerenes) or lipophilic (PCBM) behavior were synthesized in order to increase the solubility in water and organic solvents.

Fullerenes are extensively studied in a number of applications such as organic photovoltaics (Brabec et al., 2010; Kirner et al., 2014), gas storage (Gadd et al., 1999), and molecular sensing (Baena et al., 2002; Sherigara et al., 2003). In the last 30 years fullerenes, alongside CNTs, were considered among the cutting-edge nanomaterials for biomedical applications: they were proposed as oxidative damage protecting agents, photosensitizers for photodynamic therapy of cancer, antiretroviral agents and as drugs and gene delivery vectors (Bakry et al., 2007; Tykhomyrov et al., 2008; Partha and Conyers, 2009; Chen et al., 2012; Matija et al., 2013). Fullerenes also were the pioneering carbon nanomaterials investigated *in vivo* for their potential applications in the therapy of brain diseases. However, the raising concerns of their toxicity has contributed to a reduction of the interest from the biomedical scientific community.

There are conflicting reports in literature regarding their toxicity. C_60_ has been documented for ex. both to induce reactive oxygen species (ROS) mediated toxicity and to provide efficient protection from ROS damage (Johnston et al., 2010). While it is generally accepted that pristine C_60_ displays just moderate toxicity (Tokuyama et al., 1993; Partha and Conyers, 2009; Aschberger et al., 2010; Johnston et al., 2010), its covalent and non-covalent derivatives can be instead very toxic (Trpkovic et al., 2012; Sergio et al., 2013). Furthermore, toxicity of pristine C_60_ is increased by the presence of surfactants or organic co-solvents (Johnston et al., 2010) and genotoxicity has been also reported (Dhawan et al., 2006). The high affinity of C_60_ for a number of chemical species along with its ability to permeate the biological membranes allows it to convey toxicants in the cells or to interfere with metabolic processes (Sergio et al., 2013). It should be noted however that toxicity evidences show a certain degree of variability that can be ascribed, in large part, to the very different experimental conditions and toxicity assays used.

Fullerenes are able to penetrate the neuronal cell membrane both *in vitro* and *in vivo* (Yamago et al., 1995; Dugan et al., 2001). They can accumulate in several tissues and, notably, they cross the BBB (Yamago et al., 1995). Despite the general indications of cytotoxicity, on neuronal cell cultures these compounds show neuroprotective and antioxidant effects (Dugan et al., 1996, 1997; Bisaglia et al., 2000).

*In vivo*, fullerenes are the first carbon nanomaterials found to distribute in the brain after systemic administration. Biodistribution studies using a ^14^C-radiolabeled carboxylated C_60_ derivative (^14^C-C_60_) in rats after *i.v*. administration (Yamago et al., 1995) reveal that the nanomaterial rapidly spreads in several organs including brain, indicating that it is able to cross the BBB despite its high molecular weight (995 Da). No toxic effects are observed after *i.v*. administration, while toxicity is observed after intraperitoneal injection. A possible explanation for this different behavior can be that the fullerene is able to induce a consistent inflammatory response only when it is administered in a confined site at high concentrations, while direct dilution in the bloodstream suppresses this accumulation-dependent toxicity. However, as the authors of this study point out, this nanomaterial has a high lipophilicity, which translates into slow excretion kinetics and accumulation in specific organs. This raises concerns about the possible occurrence of long-term toxicity or toxicity after chronic administration since the fullerene can reach with time toxic concentrations inside specific sites.

Alike CNTs, also fullerenes have been probed for their potential therapeutic activity in the CNS, especially for ROS-scavenging purposes. The first and most studied fullerene demonstrating this property is carboxyfullerene, a C_60_ tris(malonic acid) water soluble derivative. Carboxyfullerene continuous *i.p*. administration by means of a mini-osmotic pump in transgenic mice carrying a human superoxide dismutase gene mutation related to familial amyotrophic lateral sclerosis (FALS) results in 15% delay in the appearance of FALS symptoms, and 6% increase of survival (Dugan et al., 1997). Carboxyfullerene is also able to protect nigrostriatal dopaminergic neurons against the oxidative stress generated by Iron(II) injection (Lin et al., 2001), used as a Parkinson's disease model: intracranial co-administration of the nanomaterial at low doses with Iron(II) into mice's *substantia nigra* is able to inhibit the induced ROS generation, keeping dopamine levels and dopaminergic response similar to basal values. Although the two nanomaterials are co-administered, it is unlikely that neuroprotection occurs thanks to metal sequestration or direct reduction of the metal ion operated by the fullerene, rather it is likely to act as free radical scavenger as demonstrated by EPR spectroscopy experiments (Dugan et al., 1997). Finally, in MCAO stroke model, intraventricular injection of high doses (0.3 mg/rat) of carboxyfullerene in rats brain 30 min prior to infarction is able to fully contrast the ischemia-generated ROS production, providing 83% reduction of the infarcted area (Lin et al., 2002). Nevertheless, in the latter case the authors report adverse effects such as writhing with stretching of the trunk in more than a third of the treated animals, with death occurring in the 60% of these cases. Administration of a lower (but still high) dose (0.1 mg/rat) of the nanomaterial is free of adverse effects, but has limited efficacy. Systemic administration (6 mg/kg) through the tail vein also demonstrates to be nontoxic, however it has no effect on the infarction. These results indicate that the nanomaterial displays in general acute toxicity when employed locally in the CNS at high dosage, while it can be considered reasonably safe when it is locally administered at low doses or when systemic administration is employed.

Carboxyfullerene is proposed also as neuroprotective cerebral antiaging compound: daily administration of the nanomaterial to mice (10 mg/kg/day) in drinking water is able to reduce the superoxide content in brain tissues to levels just above those of control young mice, implying that the nanomaterial is able to cross the BBB (Quick et al., 2008). An improved ability in memory behavioral tests and a 11% lifetime increase is also observed, suggesting a considerable antiaging effect exerted also to several other organs. A neuroprotective effect of carboxyfullerene after single systemic *i.p*. administration is evidenced also against *E. Coli* induced meningitis in mice (Tsao et al., 1999): although the nanomaterial has no direct antibacterial activity, preventive administration as well as post-infection treatment with carboxyfullerene (6–40 mg/kg, administered 3 times every 24 h) decreases brain inflammation by modulating the immune response and preventing the BBB leaking due to inflammation, thus delaying or partially preventing (up to 80%) mice death in a dose-dependent way and more effectively than corticosteroids. The high doses of nanomaterial injected do not cause any toxic effect in mice, thus strengthening the hypothesis that the systemic administration of this nanomaterial is particularly well tolerated.

**Figure 5:**
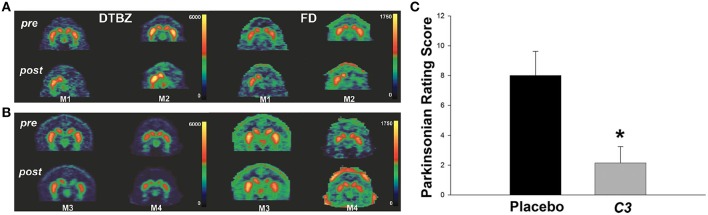
**(A,B)** Positron emission tomography (PET) brain images using from two control primates (M1, M2) and two carboxyfullerene-treated primates (M3, M4) before MPTP injection (pre) and at the end of the treatment (post). [^11^C] dihydrotetrabenazine (DTBZ) and 6-[^18^F] fluorodopa (FD) are used as probe for evaluating the nigrostriatal dopaminergic activity. As clearly visible placebo-treated animals are showing unsymmetrical distribution of tracers in the two hemispheres indicating partial loss of dopaminergic activity, while carboxyfullerene-treated animals are showing dopaminergic activity in both the hemispheres. **(C)** Parkinsonian rating score at the end of the treatment, indicating reduction of bradykinesia in carboxyfullerene (C3) treated animals with respect to animals receiving placebo. Data reported as mean + s.e.m. ^*^p = 0.007. Adapted from Dugan et al. (2014) with permission from John Wiley and Sons, Copyright (2014).

Carboxyfullerene has been also tested for the treatment of Parkinson's disease (PD) in MPTP treated non-human primates models (Dugan et al., 2014). The nanomaterial is delivered 1 week after MPTP injection by continuous systemic administration (3 mg/kg/day) using either intraperitoneal or subcutaneous osmotic pumps. Significant differences between the placebo group and animals receiving carboxyfullerene are found starting from 30 days after the beginning of the treatment, with treated animals showing motor ability improvements approaching normal values at the end of the experiment (Figure 5). This indicates that the continuous administration of carboxyfullerene is able to induce the recovery of dopaminergic neurotransmission even after the MPTP-induced neuronal death process has already begun. Moreover, despite the prolonged duration of the experiment and the continuous administration of the nanomaterial, only little evidences of toxicity are found. Results promote therefore a potential application of this nanomaterial for the cure of Parkinson's disease in humans.

Alongside carboxyfullerene, other C_60_ fullerene derivatives show neuroprotection capabilities: hexasulfobutylated-C_60_ (FC_4_S), when administered *i.v*., is able to cross the BBB and prevent oxidative damage after MCAO stroke induction (Huang et al., 2001), providing up to 67% reduction of the infarcted brain volume after reperfusion. Intracellular oxidative stress is found to be perfectly normal, while interestingly the presence of increased levels of nitric oxide (NO) suggests that FC_4_S may exert its neuroprotective action by activating specific cell signaling pathways. Furthermore, the authors report no adverse effect of the FC_4_S administration. Thanks to the possibility to use *i.v*. administration and to the absence of adverse effects, this nanomaterial therefore is able to overcome the limitations displayed by carboxyfullerene (Lin et al., 2002) in the prevention of stroke-deriving ischemic damage. However, the prevention of brain infarction damages implies that the nanomaterial has to be chronically administered and therefore, prior to envisage possible uses of this nanomaterial in therapy, long-term toxicity studies must be performed. Among noncovalent derivatives of C_60_ fullerene, its adduct with poly(vinylpyrrolidone) (C_60_-PVP) displays neuroprotective capabilities: direct injection of the nanomaterial into rats hippocampus is able to protect memory consolidation mechanisms in rats when these are treated with cycloheximide, a protein synthesis inhibitor able to impair the memory consolidation processes (Podolski et al., 2005). Results seem to indicate that the ROS-scavenging ability of the fullerene adduct is the main responsible of preventing the neuronal apoptotic response to the drug. However, although no indications of adverse effects in the CNS are provided, the choice of local cerebral administration of this therapeutic agent raises concerns regarding the possible occurrence of toxicity.

Also unfunctionalized fullerene (C_60_), in the form of water suspension of the pure nanomaterial as hydrated (C_60_HyFn), can be used for neuroprotection purposes. The administration of the nanomaterial in rats drinking water provides protection against neuronal damages deriving from chronic alcohol intake (Tykhomyrov et al., 2008). Analyses reveal that the nanomaterial is able to contrast the alcohol-induced depletion of glial fibrillary acidic protein (GFAP) in astrocytes as well as to preserve the expression of cytoskeletal proteins also in neurons and glia. No adverse effects due to the nanomaterial intake are observed. Additionally, C_60_HyFn demonstrates possible neuroprotective activity against Alzheimer disease (AD) neurodegeneration. *In vitro*, the nanomaterial is able in fact to interfere with the formation of *A*β_**25–35**_ amyloid peptide fibrils structure, resulting in the accumulation of protofibrillar structures (Podolski et al., 2007). Rats injected with the amyloid peptide rapidly develop dementia, but when even low doses (maximum 5 μg/rat) of C_60_HyFn are injected intracerebroventricular (*i.c.v*.) prior to the injection of the amyloid peptide, rats show normal cognitive abilities. Also, no evidence of nanomaterial toxicity is found (Podolski et al., 2007). A recent follow-up in this research indicates that hippocampal injection of C_60_HyFn is able to restore the cortical-hippocampal EEG interrelations disrupted by the injection in the same site of an *A*β peptide to simulate AD (Vorobyov et al., 2015). As these reports demonstrate, unfunctionalized C_60_ fullerene show neuroprotective effects when administered both GI and locally in the CNS. Differently from carboxyfullerene however, unfunctionalized C_60_ does not show toxicity when administered directly in the brain tissues. Toxicological investigations on C_60_ fullerene suggest however a potential long-term toxicity of the nanomaterial (Yamada et al., 2008). Even though no severe acute toxicity is found, the *i.c.v*. injection of C_60_ is in fact found to interfere with neurotransmitter homeostasis in rats, causing behavioral changes in the animal. Interestingly, *i.p*. injection of C_60_ does not provide alterations in the cerebral neurotransmitters levels.

The last fullerene-derived nanomaterial that showed direct neuroprotective capabilities *in vivo* is fullerenol, i.e., polyhydroxylated C_60_ (C_60_-OH). This derivative, which is already known from *in vitro* studies to display neuroprotective activity (Jin et al., 2000), and its glucosamine conjugate (GlcN-F), designed in order to add anti-inflammatory activity to the nanomaterial, demonstrate to be very good neuroprotective agents against stroke insult (MCAO induced) after systemic administration in both normotensive (WKY) and hypertensive (SHR) rats (Fluri et al., 2015). The *i.v*. administration of 0.5 mg/kg of C_60_-OH subsequent to reperfusion after MCAO results in 68% reduction and 26% reduction of the infarcted area volume (compared to control) in WKY and SHR rats respectively. The increase in C_60_-OH dosage does not provide sensible therapeutic improvements but results in the appearance of adverse effects and also of death, while the use of GlcN-F (5 mg/kg, equivalent to 0.5 mg/kg of C_60_-OH), in SHR rats provides a greater reduction of the infarcted volume with respect to C_60_-OH without evidences of toxicity. However, it is found that the *i.c.v*. injection in rats of even small doses (0.25 mg/kg) of fullerenol produces important—although transitory—toxic effects on the monoamine neurotransmission and animal behavior (Yamada et al., 2010). This raises further and more alarming concerns about the nanomaterial safety, also regarding possible adverse effects that can arise in case of accumulation of the nanomaterial in the brain also after systemic administration. It should be underlined also that the results provided by C_60_-OH are in line with those of FC_4_S (Huang et al., 2001). However, FC_4_S seems to be effective at lower doses and it is not toxic. The effectiveness of GlcN-F in reducing the extent of infarcted brain volume in SHR rats is instead a very appreciable result since it is known that hypertension has a strong detrimental effect on the prognosis after stroke. It would be interesting then to examine the neuroprotective effect of FC_4_S also in hypertensive rats in order to determine if it can be an equally effective and safer alternative to GlcN-F.

Although extensive researches have been conducted to address the intrinsic neuroprotective properties of fullerenes, there are very few reports regarding *in vivo* drug delivery and imaging applications within the CNS. Drug delivery has been probed using a C_60_ derivative having two enzymatically cleavable amantadine molecules, synthesized with the aim to create a new anti-parkinson agent that combined the pharmacological activity of amantadine with the neuroprotective activity of the fullerene (Nakazono et al., 2004): studies on Parkinson model rats demonstrate moderate activity of the fullerene drug when systemically administered at 10 mg/kg dose, while at higher doses the drug is ineffective probably because the nanodrug itself is inhibiting the enzyme deputed to the hydrolysis of the fullerene-amantadine bond. On the other hand brain tumors bioimaging using fullerenes derivatives was achieved by means of endohedral gadolinium-C_82_ fullerenol (Gd@C_82_-OH), where the paramagnetic Gd^3+^ cation is enclosed in the fullerene cage: after *i.v*. injection the nanomaterial can detect, by means of MRI, a C6 glioma tumor in rats brain (Shevtsov et al., 2014). The nanomaterial is accumulating inside the tumor and displays a higher detection efficiency than the standard contrast agents. Gd@C_82_-OH is found to be nontoxic to the animals unless high concentrations (≥12.5 mg/kg) are used, while it is able to increase their survival time, implying also a potential antitumor activity.

In summary, fullerenes demonstrate a good potential as neuroprotective agents, while their use as drug delivery vectors or imaging agents, at least in the CNS, has been just marginally explored. Most importantly fullerenes, and in particular carboxyfullerene, display a not negligible toxic profile for the CNS that however can be drastically reduced when systemic administration is preferred to local acute administration. However, neuroprotective applications require chronic administration of the therapeutic agent, and long-term toxicological data on these nanomaterials are still scarce. Despite the very good results achieved, fullerenes represent the “past” of carbon nanomaterials research. This again is due to all the concerns related to their proven accumulation in several organs, their long persistency in the body and their—in general—unpredictable toxicity. With all these serious impairments, it is not easy to say if the risk-benefit ratio will still provide opportunities for the development of these nanomaterials for biomedical applications.

## Graphene oxide and derived nanomaterials

**Figure 6:**
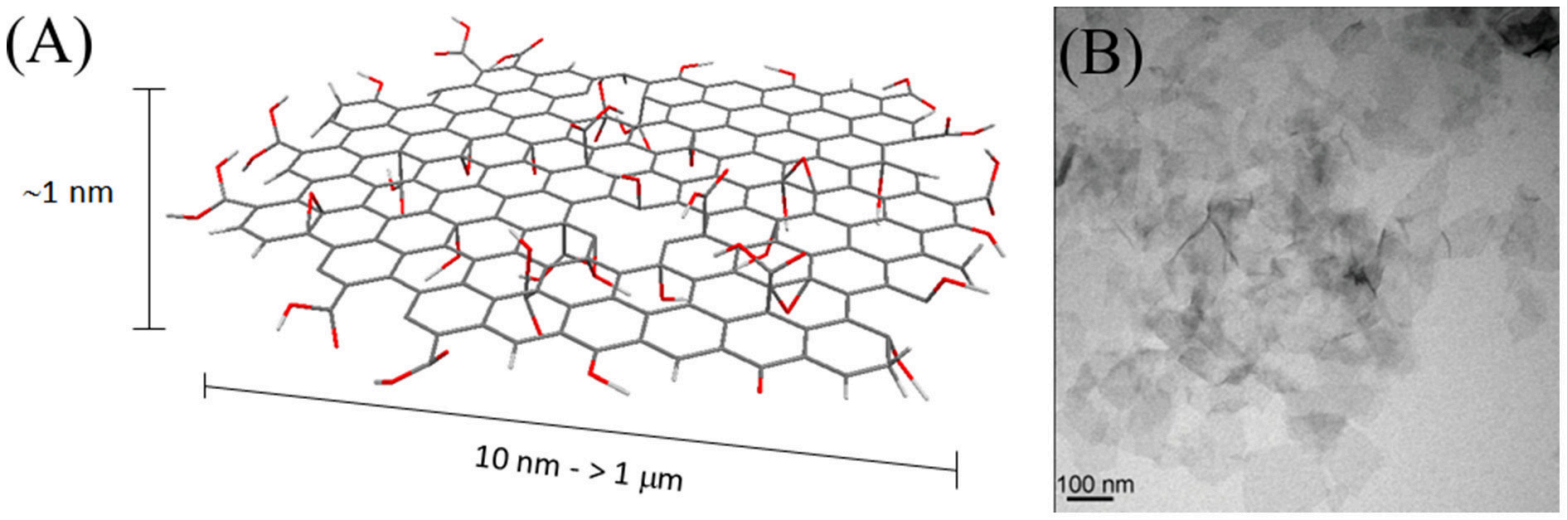
**(A)** Schematic representation of GO. The nanomaterial surface and edges are characterized by the presence of carboxyls, carbonyls, alcohols, and epoxydes. **(B)** TEM micrograph of GO sheets; adapted from Zhang L. et al. (2010) with permission from John Wiley and Sons, Copyright (2010).

Graphene is a thin layer of sp^2^-hybridized carbon atoms bonded together in a hexagonal honeycomb lattice. Its peculiar electronic properties and structure attract a lot of attention especially in the field of semiconductor technologies. Moreover, thanks to its high surface to volume ratio applications as high capacity storage material or as drug delivery system are also proposed. Graphene oxide (GO) is the most common derivative of graphene, made from the exfoliation of graphite by oxidation procedures. GO nanoparticles are usually 1 nm thick while their lateral size can span from few tens of nm to few μm (Figure 6). Albeit the synthetic procedure introduces defective sites that destroy the peculiar electronic properties of graphene, the presence of polarizable functionalities increases its stability as single free-standing layers and allows the direct further functionalization of the material. For these reasons, graphene oxide has been considered more suitable than graphene for biomedical applications. Moreover, depending on the size, composition, and degree of oxidation, GO can exhibit inherent and tunable optical absorption and emission properties, with emission wavelengths varying from NIR to blue light (Li J. L. et al., 2012; Zhu S. et al., 2012; Cao et al., 2013; Zhang X. et al., 2013).

Graphene and derived nanomaterials are intensively applied for biomedical purposes and show promising results in toxicants and tumor marker sensing, *in vitro* and *in vivo* imaging applications, drugs and nucleic acid delivery, tumor photothermal ablation, as well as stem cell differentiation substrates (Peng et al., 2010; Zhang L. et al., 2010; Huang, 2011; Kim et al., 2011; Robinson et al., 2011; Lee W. C. et al., 2011; Gollavelli and Ling, 2012; Hong et al., 2012; Li M. et al., 2012; Chung et al., 2013; Lalwani et al., 2013; Goenka et al., 2014). Their toxicity profile is, as for many other carbon nanomaterials, highly dependent on the functionalization, size and the aggregation behavior (Jastrzebska et al., 2012; Hu and Zhou, 2013; Wick et al., 2014). GO appears to be less toxic than pristine graphene, reduced graphene oxide or hydrogenated graphene, and the additional functionalization (with PEGs, aminogroups, etc.) contributes to a further reduction of the toxicity; graphene particles with few nm diameter are less toxic than larger particles; highly dispersible particles are less toxic than the aggregating ones. With respect to the other carbon nanomaterials, graphene and derivatives tend to accumulate in lungs and to reside in the organism for a longer time (Wang et al., 2010; Zhang Y. et al., 2010; Zhang X. et al., 2011; Yang et al., 2013b; Chng et al., 2014; Chwalibog et al., 2014; Kanakia et al., 2014; Seabra et al., 2014).

Recently, graphene and related materials are emerging as a convenient substrate and a powerful tool for neuronal growth and differentiation. Reports indicate that graphene is a permissive substrate for neuronal cells growth (Li et al., 2011, 2013; Park et al., 2011b; Movia and Giordani, 2012; Hong et al., 2014; Serrano et al., 2014; Tu et al., 2014; Fabbro et al., 2016) and the electrical conductivity of this material can be exploited to direct the elongation of neuronal processes in a controlled way (Li et al., 2011). Moreover, the electrical signals generated from neuronal cells can also be recorded by using graphene-based microelectrodes (Chen et al., 2011; Tang et al., 2013; Park et al., 2014). Surprisingly, it has also been shown that the physicochemical properties of this material favor the differentiation of neuronal stem cells preferentially toward the neurons fate (Park et al., 2011b; Wang et al., 2012; Akhavan and Ghaderi, 2013; Li et al., 2013). In these studies, toxicity assays show a good compatibility of graphene with neuronal cells (Chen et al., 2011; Li et al., 2011; Hong et al., 2014). In particular, reports show that graphene flakes (Zhang Y. et al., 2010) and graphene-based substrates (Hong et al., 2014; Song et al., 2014) may be even more compatible than other carbon-based nanostructures.

*In vivo* biodistribution studies reveal that GO has good potential for applications in the CNS: intravenous administration of radiolabeled GO (^188^Re-GO, 10–800 nm lateral size) in mice (Zhang X. et al., 2011) indicates that, despite most part of the nanomaterial is sequestrated by lungs, a small quantity (0.04% of injected dose) is able to cross the BBB and migrate into the brain parenchyma. Similar results are obtained by administering *i.v*. GO (0.3–1 μm lateral size) as suspension in PBS with the help of a surfactant. Remarkably, the presence of the surfactant allows to reduce lung accumulation, erythrocyte agglutination and macrophage activation (Qu et al., 2013). A further improvement has been made recently by noncovalently functionalizing GO with dextran (GO-DEX, 100–120 nm lateral size): after *i.v*. administration in mice, the nanomaterial is found to pass the BBB without exerting toxic effects in the brain and showing just minor effects in the other organs at the highest doses (>125 mg/Kg) (Kanakia et al., 2014). Interestingly, brain GO-DEX concentration is found 3 times higher 1 month after the injection with respect to 24 h after the injection, while it is almost completely cleared from all the other organs, thus indicating slow accumulation and long-term persistency of this nanomaterial in the CNS. If this can be considered a strength in view of applications as neuroprotective agents, on the other hand it raises concerns about possibilities of long-term toxicity, which however has not been explored yet.

**Figure 7:**
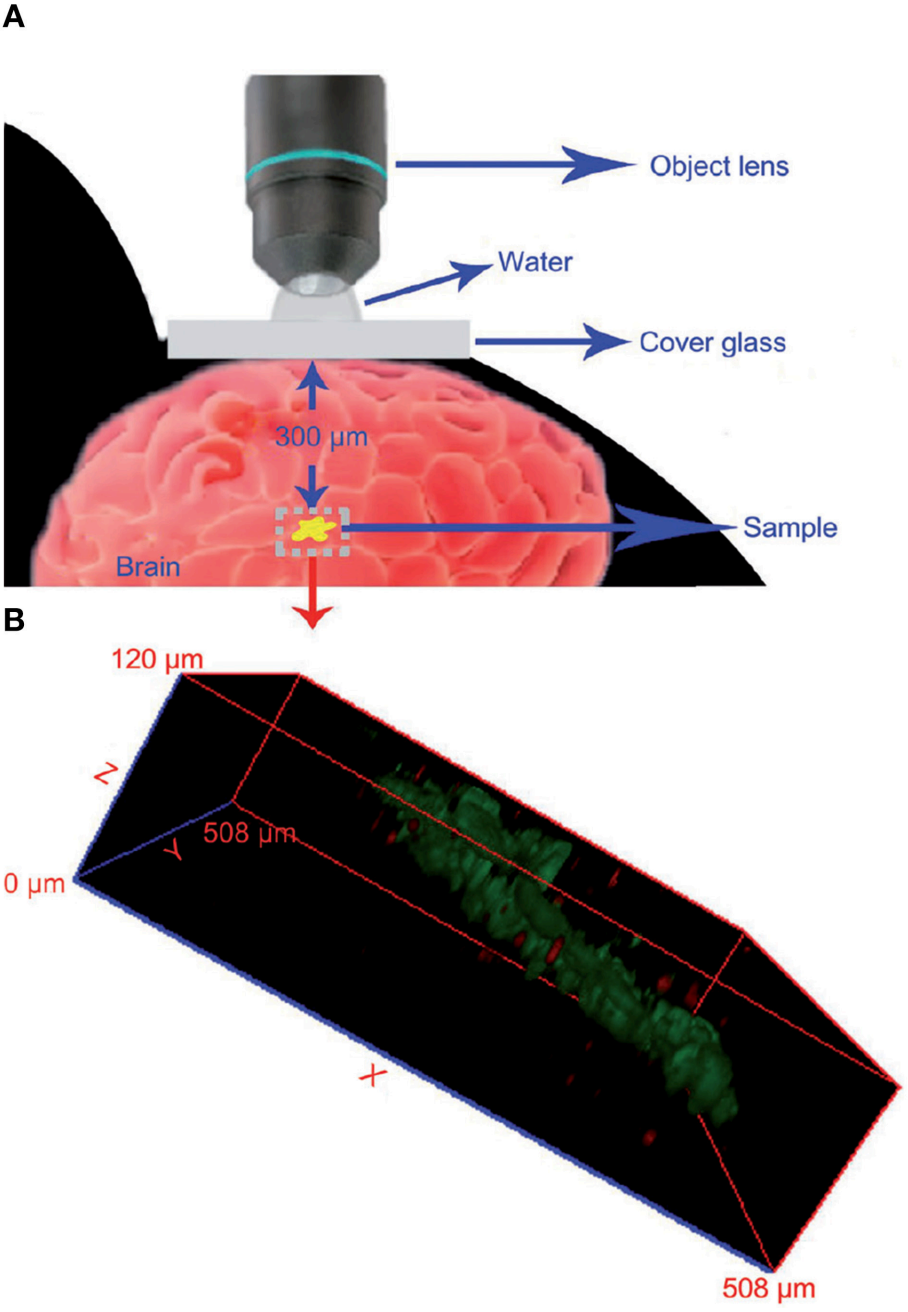
**Imaging of GO nanoparticles in a mouse brain using two-photon luminescence. (A)** Schematic representation of the experimental conditions used. **(B)** Reconstructed 3D luminescence image of GO-PEG nanoparticles inside the brain parenchyma. Reprinted from Qian et al. (2012) with permission from John Wiley and Sons, Copyright (2012).

Contrary to CNTs and fullerenes, GO and derivatives are not showing remarkable ROS scavenging capabilities *in vitro*, therefore no *in vivo* studies have been performed in order to assess their potential neuroprotective activity. Interestingly, GO and derivatives can be successfully applied for *in vivo* imaging purposes in the brain. PEG-functionalized GO (GO-PEG, 40 nm lateral size), intracranially administered in mice, can be detected thanks to its fluorescence emission properties up to 300 μm below the brain surface and its 3D distribution map in the brain parenchyma reconstructed (Figure 7) by using the two-photon imaging technique (Qian et al., 2012) in order to achieve high tissue penetration of the excitation light. Although preliminary, these results pave the way to the possible use of this nanomaterial for the imaging of brain cancerous lesions. This can be achieved firstly if GO nanoparticles are endowed of appropriate tumor-targeting functionalizations able to cause the selective accumulation of the nanomaterial inside the tumor mass. Furthermore, there is need to optimize the the nanomaterial characteristics (size, degree of oxidation), in order to shift its emission wavelength from the VIS spectral range that has poor tissue penetration, to the NIR, thus improving the imaging depth that is possible to achieve.

**Figure 8:**
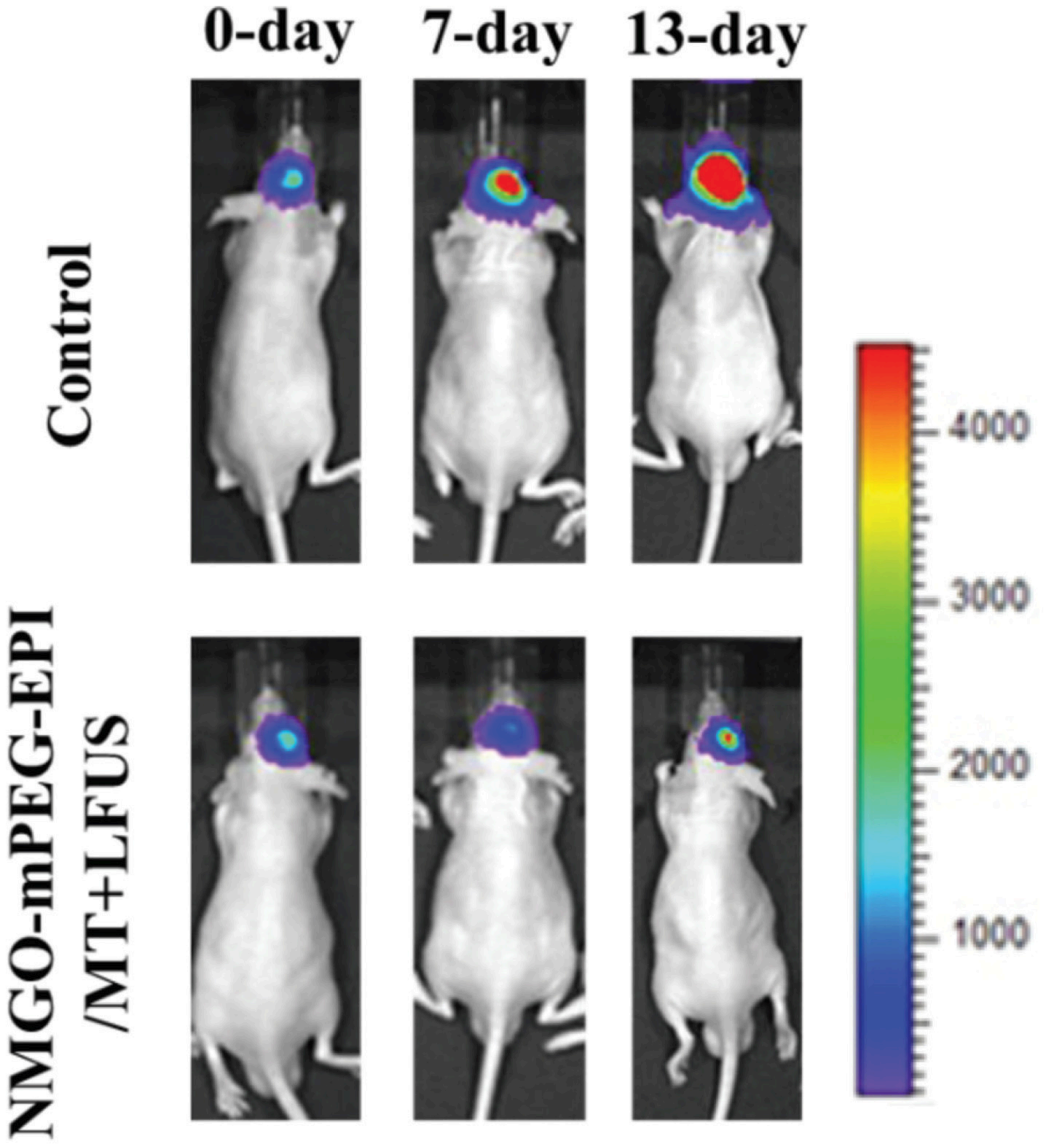
***In vivo* luminescence imaging of luminescence-labeled U87 tumor xenografted into nude mice brains**. Animals receiving the treatment consisting in administration of magnetic GO-PEG-EPI nanoparticles followed by magnetic targeting and LFUS (NMGO–mPEG–EPI/MT) show an improved tumor reduction at 7 and 13 days after the treatment with respect to control mice. Adapted from Yang H.-W. et al. (2013) with permission from John Wiley and Sons, Copyright (2013).

Although GO shows scarce tendency to reach high concentrations in cerebral tissues after systemic administration, its high specific surface area, which endows it of a high loading capacity, makes it a promising candidate also for drug delivery applications in the CNS. Strategies, such as low-invasive physical BBB opening techniques or chemical functionalization with efficient targeting moieties, can be used to overcome the low BBB permeability of GO. GO-PEG nanoparticles (120–150 nm lateral size) loaded with epirubicin (EPI), an anticancer drug, and decorated with magnetic Fe_3_O_4_ nanoparticles, can be used against U87 glioma xenographted in mice striatum (Yang H.-W. et al., 2013): after administration in the jugular vein, the nanodrug can be accumulated in the tumor mass by combining the use of low intensity focused ultrasound (LFUS), a physical BBB opening technique, and magnetic targeting. This results in a significant reduction of the tumor growth rate in the treated mice compared to control mice (Figure 8). It seems also that the use of GO and LFUS in combination is particularly effective due to the obtainment of local hyperthermia in the tumor. Magnetic GO-PEG-EPI nanoparticles are found to accumulate preferentially in the liver, from which they are completely cleared in 48 h. No organ damages or weight loss, neither *in vitro* induction of immune response is found. The relatively rapid GO clearance and the absence of acute toxicity phenomena make this nanomaterial a suitable candidate for implementing the current brain tumor therapies.

Physical BBB opening techniques combined with GO can be also used to obtain simultaneous MRI imaging, drug delivery and miRNA delivery in the CNS. GO nanoparticles (140–150 nm lateral size) grafted with Gd-DTPA and poly(amidoamine) dendrimer, and loaded with EPI and *Let-7*, a tumor suppressor miRNA (Yang H.-W. et al., 2014) can be administered by tail vein injection in mice and allowed to cross the BBB thanks to the application of focused ultrasounds (FUS). The nanomaterial is able to provide very high contrast in MRI, which can be used in order to determine and quantify the distribution of the nanometric drug delivery system inside the brain tissues. Unfortunately, the study limits the demonstration of EPI internalization by glioma cells and miRNA transfection in their nuclei only to *in vitro* experiments. Although the results provided are very positive, it will be important to demonstrate *in vivo* the therapeutic efficacy of the nanodrug, and also to obtain pharmacokinetic and toxicological data.

Also chemical derivatization with suitable targeting moieties is able to provide the nanomaterial BBB crossing capabilities, making possible to pursue efficient drug delivery in the CNS. Transferrin (Tf) functionalized GO-PEG nanoparticles (Tf-PEG-GO, 100–400 nm lateral size) are successful in delivering DOX in a brain tumor (Liu G. et al., 2013): after *i.v*. administration the nanoparticles are able to migrate from the bloodstream to a C6 glioma that has been implanted in rats striatum, where they are found significantly more concentrated than in the rest of the brain and the other tissues. Also, tumor DOX retention is increased with respect to controls. As result, Tf-PEG-GO-DOX nanoparticles can significantly delay the tumor growth and increase the rats median survival time, although no complete tumor eradication is noticed. Similarly, GO-PEG (100–300 nm lateral size) functionalized with Human Immunodeficiency Virus (HIV) Tat protein derived peptide (Tat), which increases the BBB permeability of the nanomaterial, can deliver drug molecules inside the brain tissues (Yang et al., 2015). The targeted nanovector is able to improve the perfenidone (PERF) efficacy in the treatment of subarachnoid hemorrhage, whose success is limited by the scarce BBB penetration of the drug. Photoacustic imaging demonstrates that the nanodrug is able to accumulate in the brain after *i.v*. administration and that there is a clear improvement with respect to the standard PERF therapy in the PERF-induced water content increase close to the injured site. Finally, evaluation of BBB integrity after the nanocarrier administration reveals that its structure and function are not affected by the nanoparticles.

In conclusion, the studies above reported suggest that GO and its derivatives have many properties that can make them suitable candidates for both diagnostic and therapeutic applications in the CNS: they display intrinsic fluorescence and they can diffuse inside the brain tissues, they have high loading capacity that allows them to deliver significant quantities of drugs or imaging agents inside the brain and, to date, they have not displayed toxicity toward CNS tissues yet. Unfortunately, the nanomaterial displays low BBB permeability *per se*, and functionalization with high efficiency targeting molecules or the employment of novel physical BBB opening techniques is mandatory in order to overcome this issue. We have to remark however that researches aiming to propose possible applications of GO in the CNS are relatively recent and therefore the nanomaterial has not been yet optimized (size, functionalization, dose, etc.) for the best performances in this body region. For the same reason, toxicity of GO toward CNS has not been deeply investigated, including a careful examination of GO effects both on single neuronal populations and in the whole CNS systematically evaluating the effect of size and functionalization. It is expected that the high attention given nowadays to graphene and derivatives will stimulate rapid improvements both in GO engineering for medical applications, including those involving the CNS, and in the understanding of its eventual toxic effects there.

## Nanodiamonds

**Figure 9:**
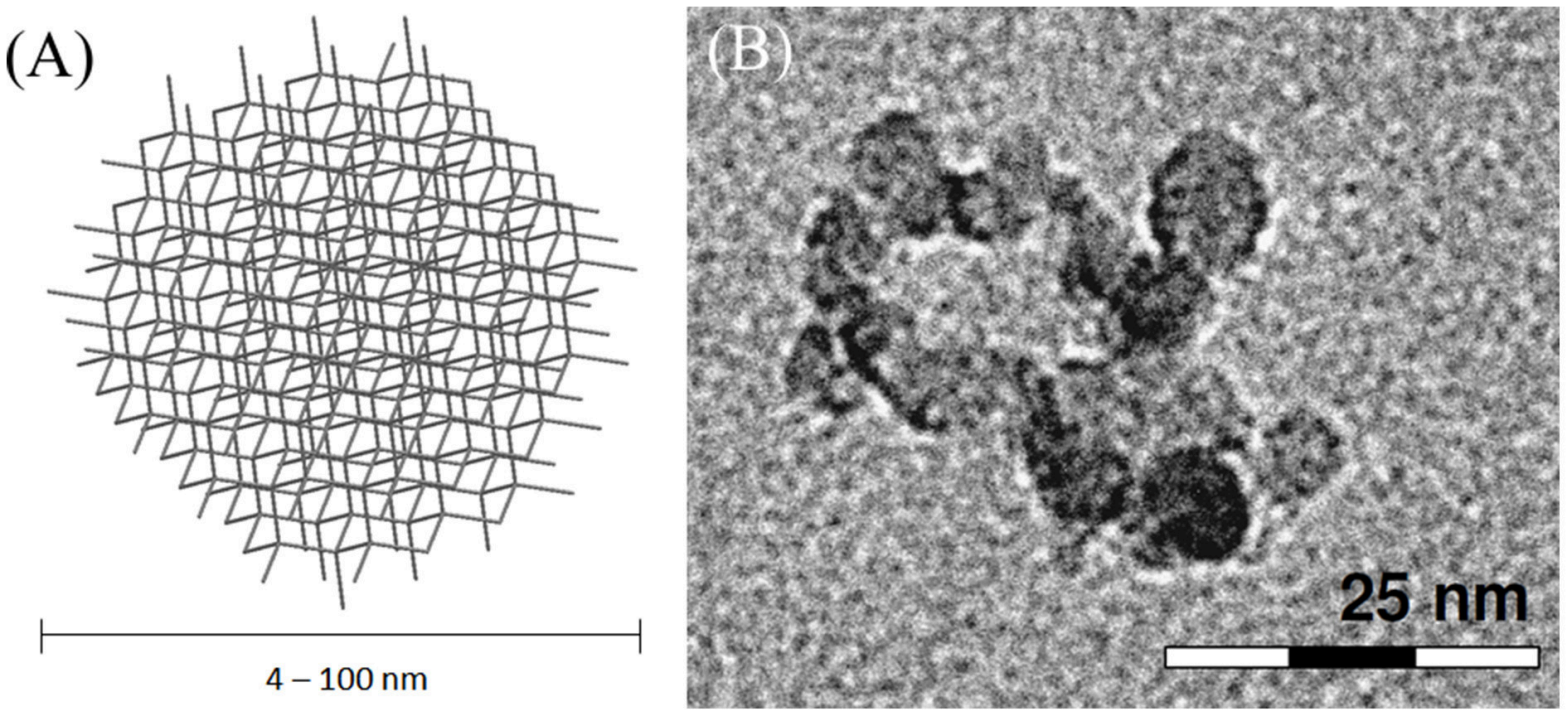
**(A)** Schematic representation of NDs. **(B)** HRTEM micrograph of ~7 nm oxidized diamond nanoparticles; adapted with permission from Rojas et al. (2011). Copiright (2011) American Chemical Society.

Nanodiamonds (NDs) are carbon particles formed by sp^3^ carbon atoms arranged in a diamond-like cubic lattice. They can be produced in several diameters, ranging from 4–5 to 100 nm (Figure 9). NDs synthesis is usually performed at high pressure-high temperature. Although several production methods were developed, the most used is detonation of TNT and nitroamines (RDX) (Galli, 2010; Mochalin et al., 2011). NDs are currently the most abundantly produced carbon nanostructures due to the number of industrial applications where they are employed, especially for the lubricants and polishing industry and as part of novel high-performance nanocomposite materials (Mochalin et al., 2011).

Nanodiamonds surface is highly reactive and can be easily functionalized, as well as passivated (Liu et al., 2008; Vaijayanthimala and Chang, 2009; Chen et al., 2010; Rojas et al., 2011). Furthermore, by irradiating nanodiamonds with high-energy particles and subsequent annealing, it is possible to create nitrogen-vacancy centers that render the nanodiamond particles highly fluorescent in the VIS range (500–800 nm, with peak emission at 680 nm) (Fu et al., 2007; Chang et al., 2008; Vaijayanthimala et al., 2012; Hegyi and Yablonovitch, 2013; Bartelmess et al., 2015c). Recently, they have started to be probed for possible biomedical applications like bioimaging (Vaijayanthimala and Chang, 2009; Hui et al., 2010; Hegyi and Yablonovitch, 2013; Perevedentseva et al., 2013), drug delivery and nucleic acid delivery (Xing and Dai, 2009; Chen et al., 2010; Zhu Y. et al., 2012; Perevedentseva et al., 2013), also exploiting the functionalization with targeting molecules for improved selectivity (Zhang X.-Q. et al., 2011; Fu et al., 2012). In view of their highly biocompatibility, nanodiamonds are one of the most promising carbon nanomaterials in this field (Schrand et al., 2007a,b, 2009; Perevedentseva et al., 2013; Monaco and Giugliano, 2014). When administered *in vivo*, nanodiamonds accumulate in the liver, in the spleen, and in lymphnodes (Yuan et al., 2009; Vaijayanthimala et al., 2012). Neuronal cells cultured on a surface of nanodiamonds reveal cell growth and electrophysiological properties comparable to neurons grown on classical supports (Thalhammer et al., 2010; Monaco and Giugliano, 2014; Edgington et al., 2013; Hopper et al., 2014). Nanodiamonds are internalized by various cell types, likely by chlatrin-based endocytosis (Liu K.-K. et al., 2009; Zhang X.-Q. et al., 2011), with limited or no cytotoxic effects being reported. Similar results are observed in neuronal cells (Hsu et al., 2014; Huang et al., 2014).

Despite the encouraging results both *in vitro* and *in vivo* for biological applications, *in vivo* applications of NDs in the CNS are still in their early days. To date only one report suggests the possible use of NDs for therapeutic applications in the CNS: CED (convection-enhanced delivery, an experimental high efficiency intracranial delivery system) of DOX-loaded NDs (4–8 nm) is found to provide efficient treatment of different aggressiveness gliomas xenographted in mice striatum (Xi et al., 2014). The treatment allows to extend mice survival (with respect to DOX treatment) 1.4 times in the case of the most aggressive tumor and 1.8 times in the case of the less aggressive one. Notably, in the latter case tumor is eradicated in 3 out of 5 mice, while all mice treated with non-conjugated DOX die. Experiments performed using healthy mice indicate that, while the intracerebral injection of a DOX solution cause the drug to rapidly spread in the whole brain producing tissue damage and brain edema, the use of NDs-DOX and CED allows the therapeutic agent to be confined in the injection site, reducing its toxic effects on the surrounding tissues and increasing the concentration of the drug at the injection site. Furthermore, while DOX is rapidly excreted from the brain, NDs-DOX display a much lower clearance.

Alongside the possible applications of NDs in the CNS are being suggested, also toxicological studies in this body region start to be performed. Available data indicate that 100 nm fluorescent NDs injected in mice hippocampus do not produce any relevant effect neither on mice body weight, food or water intake, nor on mice behavior in a *novel object recognition* test, which should reveal eventual hippocampal damages (Huang et al., 2014). Interestingly, the same NDs have shown *in vitro* a concentration-dependent negative role in neuronal morphogenesis, although this effect seems due to a physical impairment of growth cones and not to the interference with the cytoskeletal proteins, as on the contrary it has been often evidenced for non-carbon nanoparticles (Tay et al., 2014). It is possible to hypothesize that the 3D environment and the presence of glial cells in the living tissue is drastically limiting the nanomaterial effects on growing neurons.

Given the low amount of data available, it is difficult to draw conclusions regarding the possibility of a successful application of nanodiamonds in brain science. The exclusive use of *in situ* delivery methods in the CNS raises the question if diamond nanoparticles can cross the BBB and therefore if they are suitable for applications in drug delivery or imaging in the brain. However, the possibility to display bright and photostable fluorescence, the encouraging results obtained *in vitro*, their ability to provide efficient and prolonged delivery of a drug while confining its site of action in a limited space and the absence of reports indicating relevant toxicity of the nanomaterial toward neuronal cells suggest that NDs may give precious contributions to the diagnosis and therapy of CNS diseases. It should be underlined again that the nanomaterial is in its early years of development for biomedical applications, especially in the neurosciences field. We hope that, as in the case of GO, suitable tailoring of the nanomaterial chemical, morphological and physical properties will help to overcome its current limitations.

## Carbon nanohorns and carbon nanofibers

**Figure 10:**
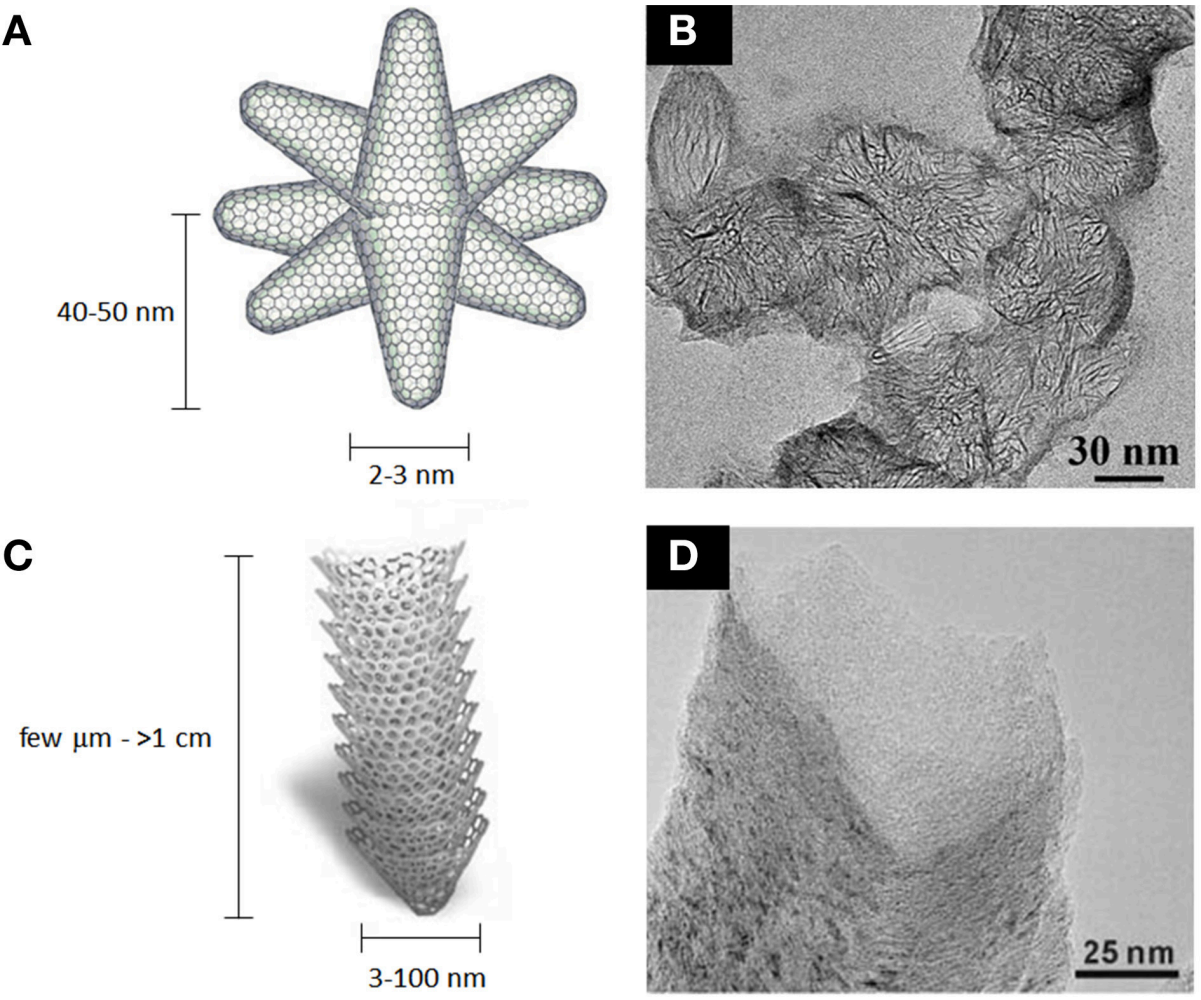
**Schematic representation of SWCNHs (A) and stacked-cup CNFs (C). (B)** TEM micrograph of ~80 nm SWCNHs peapods. **(D)** TEM micrograph of stacked-cup CNFs. **(A,B)** adapted from Voiry et al. (2015) with permission from The Royal Society of Chemistry. **(C,D)** adapted from Sato et al. (2005) with permission from The Royal Society of Chemistry.

Single-wall carbon nanohorns (SWCNHs) are relatively unexplored carbon nanomaterials, especially in biological studies. They are structurally similar to carbon nanotubes, however the continuous graphitic surface is arranged in a conical shape with a closed tip. They are usually 40–50 nm long and 2–3 nm wide, and they commonly assembly into 80–100 nm spherical aggregates (Iijima et al., 1999; Zhu and Xu, 2010; Figure 10). SWCNHs have been functionalized either covalently and noncovalently using the synthetic strategies developed for CNTs and graphene (Tagmatarchis et al., 2006; Cioffi et al., 2007; Pagona et al., 2007; Voiry et al., 2015). They find possible applications as gas storage materials (Adelene Nisha et al., 2000; Bekyarova et al., 2003; Yang et al., 2005; Sano et al., 2014), as supports for metal catalyst nanoparticles (Yoshitake et al., 2002; Kosaka et al., 2009), as electrode materials and as components of photovoltaic devices (Vizuete et al., 2010; Lodermeyer et al., 2015). Among biomedical applications, biomolecule sensing (Valentini et al., 2014), MRI imaging (as support) (Miyawaki et al., 2006), photodynamic and photothermal therapy of cancer (Zhang et al., 2008; Whitney et al., 2011; Chen et al., 2014) as well as drug and gene delivery (Murakami et al., 2004; Ajima et al., 2005, 2008; Guerra et al., 2014; Ma et al., 2014; Zhao Q. et al., 2015) are successfully achieved both *in vitro* and *in vivo* by SWCNHs.

Although SWCNHs are structurally similar to CNTs, their synthesis is metal-free, therefore no toxic effects due to metal contaminants are possible. However toxicity reports are conflicting: in some studies SWCNHs are found to be biocompatible *in vitro* as well as *in vivo* even at high doses despite their accumulation in several tissues like lung, spleen and liver (Lynch et al., 2007; Miyawaki et al., 2008; Tahara et al., 2011), while other reports demonstrate their toxicity toward macrophages even at low doses (Yang M. et al., 2014). Toxicological reports regarding carbon nanohorns *in vivo* and *in vitro* are very limited however, and it is not possible to draw clear conclusions basing on state of the art literature, although the relative higher abundance of studies indicating the presence of just low and transitory toxicity suggests their compatibility with living tissues and organs.

Unfunctionalized SWCNHs are reported to be uptaken by mammalian cells, even if in a negligible amount (Isobe et al., 2006; Zhang M. et al., 2012), while when opportunely functionalized they can efficiently penetrate target cells, potentially allowing higher selectivity than other nanoparticles (Zhang M. et al., 2012; Li N. et al., 2015) and showing good carrier properties (Tahara et al., 2011).

To date only one report describes the successful delivery of SWCNHs in the brain. SWCNHs peapods, functionalized with CdSe/ZnSe quantum dots (QDs), encapsulating Gd_3_N@C_80_ fullerenes and delivered to U87 tumor bearing mice by CED intratumoral infusion (Zhang et al., 2010c), enable tumor imaging either *in vivo* by MRI (thanks to Gd^3+^) and *ex vivo* by confocal microscopy (thanks to QDs). Data demonstrate also that SWCNHs can be retained inside the tumor for at least 3 days. Although this study indicates SWCNHs as a possible brain drug delivery nanoplatform, further reports aiming to determine the *in vivo* biodistribution of SWCNHs demonstrate that they are not able to cross the BBB (Miyawaki et al., 2009; Tahara et al., 2011). This precludes the SWCNHs to be delivered in the brain by *i.v*. administration, leaving the more dangerous and complicated intracranial administration as the only feasible option available at the moment.

Carbon nanofibers (CNFs) are tubular carbon nanostructures, with diameters in the range of 3–100 nm and lengths that can also exceed 1 cm (De Jong and Geus, 2000; Figure 10). They are essentially made of assembled curved graphitic layers arranged in different ways to form long fibers, often hollow. They are usually synthesized using CVD methods employing metal catalysts (De Jong and Geus, 2000), or from electrospun polymer fibers carbonization (Inagaki et al., 2012). Their surface can be functionalized (Klein et al., 2008; Wang and Lin, 2008) or furthermore graphitized by thermal treatment (Ramos et al., 2013). Since their first discovery in the early 50's, these materials have been tested for several applications, like catalysis and energy storage (Rodriguez et al., 1994; Ji and Zhang, 2009; Wang K. et al., 2009; Duan et al., 2015), as well as for the preparation of many composite materials (Hammel et al., 2004). Moreover, they are used as support material for biomolecules sensing (Baker et al., 2006; Wang and Lin, 2008; Huang et al., 2010; Rand et al., 2013; Lim and Ahmed, 2015), gene delivery (McKnight et al., 2003) and in regenerative medicine (Webster et al., 2004; Tran et al., 2009). As for CNTs, several concerns regarding their toxicity have been advanced (Sato et al., 2005; Castranova et al., 2013), pointing out also their non-biodegradability and their asbestos-like accumulation in lungs.

Carbon nanofibers are proposed as coating materials for neural prosthetic devices, as they show good compatibility with neuronal cells and demonstrate to favor neuronal *vs*. glial/astrocytic proliferation (Webster et al., 2004; Tran et al., 2009). Also, carbon fibers can be used to build free-standing vertically aligned arrays that allow to support and organize the neuronal cells growth providing mechanical, chemical and electrical cues at the subcellular scale. They can be also employed to produce microelectrode arrays with possible applications for *in vivo* signal detection and manipulation (McKnight et al., 2006; Nguyen-Vu et al., 2007; de Asis et al., 2009; Zhang H. et al., 2012; Vitale et al., 2015).

Despite the longstanding experience on these nanomaterials and the deep knowledge of the nanofiber-neuron interface for the preparation of efficient microelectrodes, *in vivo* experiments on their possible application for the treatment of CNS injuries and diseases are limited to just one example (Moon et al., 2012). In this report CNFs impregnated with subventricular stem cells were employed to promote neuroregeneration after MCAO-induced stroke, evaluating also the differences between “hydrophobic” CNFs (i.e., thermally graphitized, HP-CNFs, 100 nm × < 5 μm) and “hydrophilic” CNFs (i.e., untreated CNFs, HL-CNFs, 60 nm × < 5 μm) after their injection in the lesion site. The animals receiving the CNF-based treatment show reduction of the infarcted volume as well as recovery of motor and somatosensory activity, with HP-CNFs treated animals showing moderately better performances. Distribution analysis of stem cells in the brain tissues indicates that while unsupported stem cells are migrating all over the infarcted area, CNFs-supported cells localize near the corpus callosum (HL-CNFs) or the striatum (HP-CNFs). Notably, HP-CNFs are able to promote the stem cells differentiation into neurons, to induce the formation of synapsis and to reduce the astrocytes and microglia recruitment with superior efficiency with respect to HL-SWCNTs and unsupported cells. These data indicate that CNFs are optimal support material for neuronal tissue regeneration, and that a lower surface wettability is also playing a key role in promoting the stem cell differentiation toward the neuronal fate.

Although the two studies indicate that both SWCNHs and CNFs can play a role as support materials for imaging or delivery applications, they both do not display BBB crossing capabilities and require *in situ* administration. Since their very big size it is unlikely that also advanced physical BBB opening techniques or chemical functionalization can help in this sense. Due to the limited possibilities of use alongside the indications of possible toxicity, few efforts have been dedicated to identify their possible applications in the diagnosis and cure of CNS diseases. However, when used as electrodes or as part of composite nanostructures, CNFs are found to be excellent materials for promoting neuronal stimulation and growth, showing also no local toxicity. While it is likely that SWCNHs applications in the CNS will not be further explored, it is expected that CNFs will have an important role in the development of neuronal recording and stimulating devices as well as in neuroregeneration applications.

## Carbon dots

**Figure 11:**
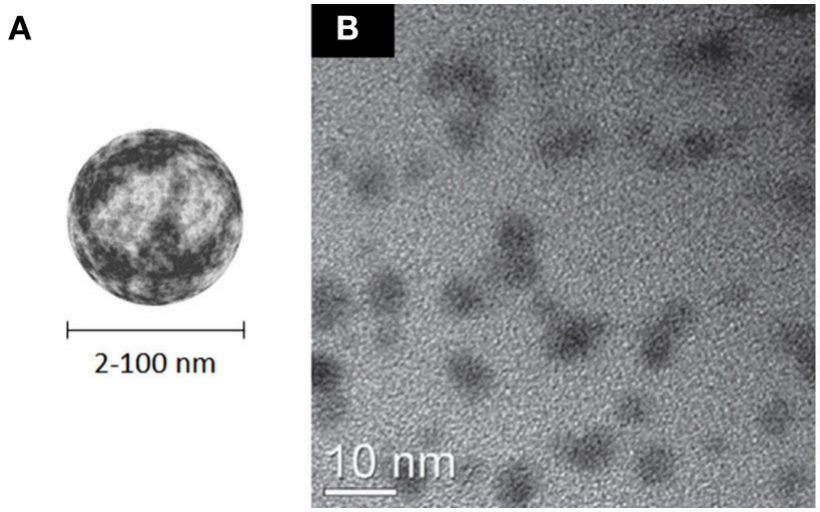
**(A)** Schematic representation of CDs. **(B)** HRTEM micrograph of 4–7 nm carbon dots produced by carbonization of chitosan; adapted from Yang et al. (2012) with permission from The Royal Society of Chemistry.

Carbon dots (CDs), are a recently discovered class of quasispherical carbon-based nanomaterials (Xu et al., 2004) which essentially combine the presence of an amorphous or nanocrystalline (Csp^3^) core and a graphitic or turbostratic (Csp^2^) shell (Figure 11). Many strategies have been developed for the synthesis of these materials, either using top-down and bottom-up approaches; however, the industrial scalability of their production is still difficult to date (Baker and Baker, 2010; Li H. et al., 2012; Lim et al., 2015; Zhang and Yu, in press). Their peculiar properties are exploited in photocatalysis (Fernando et al., 2015), electrocatalysis (Shen et al., 2015), as sensitizers for solar cells (Briscoe et al., 2015), as well as for sensing applications (Zhao A. et al., 2015). Due to their high intrinsic fluorescence which can span from the VIS to the NIR (Li H. et al., 2012; Strauss et al., 2014), CDs are considered particularly appealing for bioimaging applications (Cao et al., 2012; Liu Q. et al., 2013; Luo et al., 2013; Ruan et al., 2014; Zhang and Yu, in press), although the general excitation wavelength dependence of fluorescence emission (Sun et al., 2006; Liu et al., 2007; Qiao et al., 2010; Qu et al., 2012) can lead to artifact when they are used in combination with other luminescent probes. Depending on the synthetic strategy adopted, they already expose on their surface functional groups that allow surface passivation with biocompatible polymers and to graft additional relevant molecules (Li et al., 2010; Liu et al., 2012; Wu et al., 2013). Finally, molecules like anticancer drugs and nucleic acids can be noncovalently loaded on their surface, allowing to use these nanomaterials for delivery purposes (Lai et al., 2012; Liu et al., 2012). Among all the carbon nanomaterials described so far, carbon dots seem to display the highest biocompatibility (Zhao et al., 2008; Yang et al., 2009a,b; Li et al., 2010; Liu et al., 2010; Chandra et al., 2011; Wang Y. et al., 2011; Tao et al., 2012; Qian et al., 2014; Ruan et al., 2014). One important contribution to this effect seems to be the high density of charged groups on their surface, which provides high stability of their suspensions in water and biological fluids.

Several authors report that carbon dots penetrate cell lines when applied *in vitro* (Qiao et al., 2010; Liu C. et al., 2011; Wang F. et al., 2011; Yang et al., 2012). No toxicity is observed in various studies conducted on cell lines (Liu R. et al., 2009; Yang et al., 2009b; Wang F. et al., 2011) and on animals (Qiao et al., 2010; Tao et al., 2012). However, a recent report indicates that these nanoparticles could interfere with exocytotic mechanisms, and therefore hamper the normal neuronal and brain functions (Borisova et al., 2015). Since the effect of CDs on cellular biochemistry has not been completely unraveled, caution should be used when investigating these nanomaterials for possible clinically relevant applications.

Given their recent discovery, only few studies have been probing CDs in the CNS in view of a potential use in the diagnosis and therapy of CNS diseases. To date, *in vivo* evidences fostering the CDs use in this field derive only from biodistribution studies. Interestingly, the CDs used in these studies exhibit very good BBB crossing capabilities and a strong preference to accumulate in the brain over the other organs although they were not endowed of specific functionalizations: 100 nm fluorescent CDs, prepared *via* the inexpensive and efficient pyrolysis of a glucose and glutamic acid mixture, demonstrate to be efficiently uptaken by cerebral tissues after *i.v*. administration in mice (Qian et al., 2014). Epifluorescence imaging, made possible thanks to the CDs bright fluorescence emission, reveals that they can readily cross the BBB after systemic injection and diffuse in the brain tissues, where they reach the highest concentration within 1 h. *Ex vivo* imaging of brain slices indicates that these carbon dots are predominantly accumulating at the cortex surface, in the hippocampus and in the ventricles. Authors hypothesize that the presence of still intact glucose and glutamine molecules on the CDs surface endows the nanoparticles of “CNS-targeting” capabilities. From the available epifluorescence images, the nanomaterial does not show to diffuse in other specific body regions apart from the brain and the blood. Interestingly, the nanomaterial is also rapidly cleared from the CNS. *In vitro* studies demonstrate that CDs dispersions in plasma have high stability, they have good hemocompatibility and they are just moderately cytotoxic for brain endothelial cells only at very high concentrations. In summary the provided *in vivo* data, although referring only to early timepoints, suggest that the nanomaterial has an adequate safety profile for biomedical applications in the CNS.

**Figure 12:**
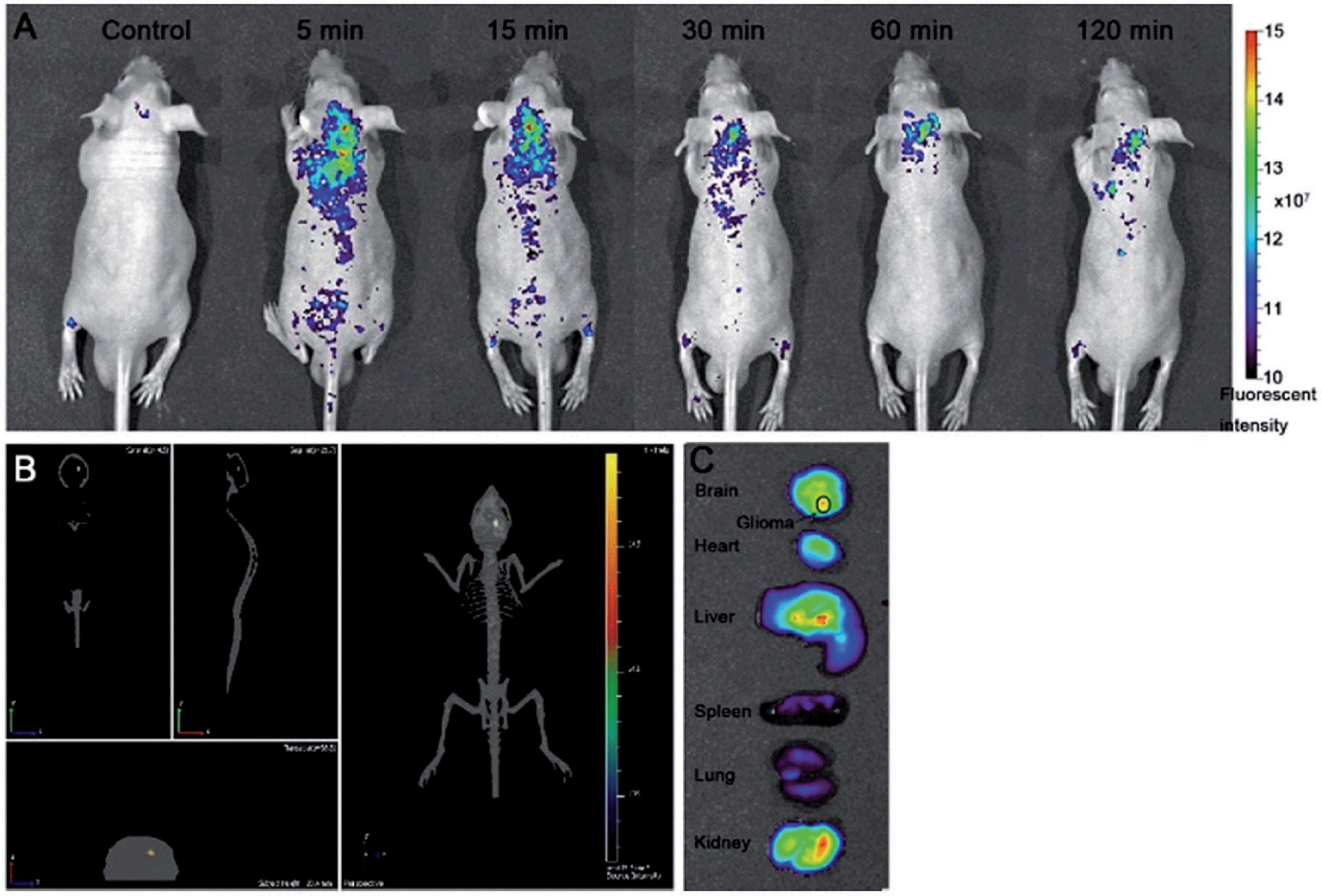
***In vivo* and *ex vivo* imaging of glioma bearing mice after administration of 3–4 nm glycine-derived CDs. (A)** Epifluorescence imaging of CDs distribution after *i.v*. injection, showing the rapid accumulation inside the glioma and the fast excretion of the nanoparticles after few hours. **(B)** 3-D reconstruction of CDs distribution 2 h after the injection, confirming the localization in the brain. **(C)** *Ex vivo* fluorescence imaging of main organs 2 h after CDs injection, indicating high accumulation in liver and kidney but also a good retention inside the glioma. Reproduced from Ruan et al. (2014) with permission from The Royal Society of Chemistry.

Also 3–4 nm glycine-derived CDs are able to cross very efficiently the BBB and accumulate in the brain. Moreover, they are able to target a human glioma tumor xenographted in mice brain (Ruan et al., 2014). Epifluorescence imaging indicates that they display a maximum brain uptake just 5 min after the tail vein injection, they strongly localize inside the tumor mass and they are also rapidly cleared. Systemically, they are also distributing in liver, kidneys and hearth (Figure 12). *In vitro* hemolysis, plasma stability and cytotoxicity studies indicate a very high biocompatibility of this nanomaterial. Although these CDs display fast and consistent accumulation inside the tumor, their potential use as vectors for delivering antitumor drugs in the CNS is not suggested at the moment because of their fast excretion form the tumor lesion and their accumulation in the heart, which is a known target of anticancer drugs toxicity.

Despite their fast elimination from brain tissues, CDs represent an excellent starting point for the development of novel diagnostic and therapeutic systems against CNS pathologies thanks to their spontaneous BBB crossing capabilities, which seem not to depend from the nanomaterial size. Moreover, these nanomaterials are in the very early stages of development for biomedical applications: opportune chemical modifications with molecules able to increase their plasma circulation time and/or with targeting moieties will be able to improve their retention in the brain and to allow this way to exploit them for applications like tumor therapy. A deep toxicological evaluation of their effects in the CNS in particular but also in the whole body however must be undertaken since current data, albeit very promising, are not sufficient to draw clear conclusions.

## Conclusions and perspectives

Carbon nanomaterials are often proposed as optimal candidates for neurobiomedical applications because of their properties which include low toxicity, high mechanical strength, high thermal and electric conductivity, and, in some cases, intrinsic fluorescence. The collection of studies and results presented in this review also demonstrates that these materials can interface with cerebral tissues, and that they can perform imaging, neuronal growth, neuroprotection and drug delivery tasks with high efficiency. Moreover, the wide variety of available carbon nanomaterials offers numerous possibilities for tailor-made actions and performances within the CNS (Table 1).

**Table 1:** **Comparison of the experimental settings and main features of the different carbon nanomaterials**.

**Carbon nanomaterial**	**Chemical modifications**	**Application**	**Animal model**	**Administration route and dose**	*****In vivo*** toxicity**	**References**
SWCNTs	^13^C enriched + Tween 80 (1%)	Biodistribution analysis	Mouse	i.v. 200 μg	Moderate (lungs, liver)	Yang et al., 2007
	−NH_2_	Neuroprotection	MCAO stroke model rat	i.c.v. 40 ng	No	Lee H. J. et al., 2011
	PEG	Neuroregeneration	Spinal cord injury model rat	Lesion site 0.025 to 2.5 μg	No	Roman et al., 2011
	PEG	Toxicity analysis	Rat	i.cr. 0.5–1.0 μg	Yes	Dal Bosco et al., 2015
				i.cr. 2.1 μg	No	
	Acetylcholine	Drug delivery	Mouse	g.i. 25 mg/kg	N.a	Yang Z. et al., 2010
	PEG oligonucleotide (CpG)	Drug delivery	Glioma-bearing mouse	i.cr. 7.5 μg	No	Zhao et al., 2011
MWCNTs	PF-127 coating	Toxicity assessment	Mouse	i.cr. 30–150 ng	No	Bardi et al., 2009
	[^111^In]DTPA	BBB crossing analysis	Mouse	i.v. 50 μg	N.a.	Kafa et al., 2015
	−${\text{NH}}_{3}^{+}$ + siRNA	Neuroprotection	Endothelyn-1 stroke model rat	i.cr. 0.5 μg	No	Al-Jamal et al., 2011
	Oxidation PEG doxorubicin angiopep-2	Targeted drug delivery	Glioma-bearing mouse	i.v. 1.9 and 6.3 mg/kg	Cardiac, lower than DOX	Ren et al., 2012
	−${\text{NH}}_{3}^{+}$	Evaluation of internalization and inflammatory potential	Mouse	i.cr. 500 ng	Weak transient infl.	Bardi et al., 2013
	Shortenting+ oxidation+ −${\text{NH}}_{3}^{+}$				Moderate transient infl.	
C_60_ fullerene	–	Neuroprotection	Rat	g.i. 3 μg/kg/die	No	Tykhomyrov et al., 2008
	–	Recovery of neuronal functions	Aβ amyloid AD model rat	i.cr. 5μg	No	Podolski et al., 2007
	–	Toxicity analysis	Rat	i.c.v. 0.25 mg/kg	Yes (moderate)	Yamada et al., 2008
				i.p. 0.25 mg/kg	No	
	Carboxylic acid (^14^C-labeling)	Toxicity analysis	Rat	i.p. 500 mg/kg	Yes	Yamago et al., 1995
			Biodistribution analysis	i.v. 0.47 mg/rat (est)	No	
	Tris (malonic acid) (carboxyfullerene)	Neuroprotection	ALS model mouse	i.p. (cont.) 15 mg/kg/day	No	Dugan et al., 1997
	Tris (malonic acid) (carboxyfullerene)	Neuroprotection	Fe^2+^- induced PD model mouse	i.cr. 3.7 μg	No	Lin et al., 2001
	Tris (malonic acid) (carboxyfullerene)	Neuroprotection	MCAO stroke model rat	i.c.v. 0.1 mg	No	Lin et al., 2002
				i.c.v. 0.3 mg	Yes (severe)	
				i.v. 6 mg/kg	No	
	Tris (malonic acid) (carboxyfullerene)	Neuroprotection	Mouse	g.i. 10 mg/kg/day	No	Quick et al., 2008
	Tris (malonic acid) (carboxyfullerene)	Neuroprotection	Mouse	i.p. 6–40 mg/kg/day, 3 day adm.	No	Tsao et al., 1999
	Tris (malonic acid) (carboxyfullerene)	Recovery of neuronal functions	MPTP-induced PD model monkey	i.p, s.c. (cont.) 3 mg/kg/day	Low	Dugan et al., 2014
	Hexa (sodium butylsulfonate)	Neuroprotection	MCAO stroke model rat	i.v. 0.1–100 μg/kg	No	Huang et al., 2001
	PVP coating	Recovery of neuronal functions	Cycloheximide memory impaired rat	i.cr. 1.7 μg	No	Podolski et al., 2005
	−(OH)_24_ (fullerenol)	Neuroprotection	MCAO stroke model normotensive and hypertensive rats	i.v. 0.5 mg/kg	No	Fluri et al., 2015
				i.v. 1.0–2.0 mg/kg	Yes (severe)	
	−(OH)_24_ (fullerenol) + glucosamine	Neuroprotection	MCAO stroke model normotensive and hypertensive rats	5 mg/kg (eq to 0.5 mg/kg fullerenol)	No	
	−(OH)_24_ (fullerenol)	Toxicity analysis	Rat	i.c.v. 0.25 mg/kg	Yes (severe)	Yamada et al., 2010
	Amantadine	Recovery of neuronal functions	Haloperidol-induced PD model rat	i.p. 10 mg/kg	No	Nakazono et al., 2004
Gd@C_82_ fullrene	Gd^3+^ endohedral −(OH)_n_	Tumor imaging	Glioma-bearing rat	i.v. ≤ 9.7 mg/kg	No	Shevtsov et al., 2014
				i.v ≥12.5 mg/kg	Yes	
GO	^188^Re-labeled	Biodistribution analysis	Mouse	i.v. 1–10 mg/kg	No	Zhang X. et al., 2011
	–	Biocompatibility analysis	Mouse	i.v. 100μg	No	Qu et al., 2013
			Tween 80 coating	i.v. 200μg	No	
	Dextran coating	Biocompatibility analysis	Mouse	i.v. < 125 mg/kg	No	Kanakia et al., 2014
				i.v. ≥125 mg/kg	Yes (low)	
	PEG	Imaging	Mouse	i.cr. 40 ng	No	Qian et al., 2012
	PEG + Fe_3_O_4_ NPs +epirubicin	Targeted drug delivery	Mouse	i.v. + LFUS 0.5 μg	No	Yang H.-W. et al., 2013
	Gd-DTPA + PAMAM + epirubicyn + miRNA (Let-7)	Imaging drug delivery gene delivery	Mouse	i.v. dose n.a.	No	Yang H.-W. et al., 2014
	PEG + transferrin + doxorubicin	Targeted drug delivery	Glioma-bearing rat	i.v. 5.6 mg/kg	No	Liu G. et al., 2013
	PEG + Tat-peptide + perfenidone	Drug delivery	Subarachnoid hemorrhage model mouse	i.c.v. 20 μg.	No	Yang et al., 2015
NDs	Doxorubicin	Drug delivery	Glioma-bearing mouse	i.cr. 30 μg	No	Xi et al., 2014
	–	Toxicity analysis	Mouse	i.cr. 1 μg	No	Huang et al., 2014
SWCNHs	QDs + Gd_3_N@C_80_	Imaging	Glioma-bearing mouse	i.cr. dose n.a.	No	Zhang et al., 2010c
CNFs	Impregnated with stem cells	Neuroregeneration	MCAO stroke model rat	i.cr. dose n.a.	No	Moon et al., 2012
	Graphitized + impregnated with stem cells			i.cr dose n.a.	No	
CDs	–	Imaging	Mice	i.v. 100 mg/kg	No	Qian et al., 2014
	–	Imaging	Glioma-bearing mouse	i.v. 100 mg/kg	No	Ruan et al., 2014

*g.i., gastrointestinal; i.cr., intracranial; i.p., intraperitoneal; i.v., intravenous; i.c.v., intraventricular; s.c., subcutaneous*.

Carbon nanotubes, amongst carbon nanomaterials, are the most studied *in vivo* for possible applications in the diagnosis and cure of brain diseases. CNTs display good compatibility with brain tissues, neuroprotective effects against stroke-induced neurodegeneration, drug and nucleic acids delivery capabilities as well as support capabilities for neuroregeneration applications. Despite these promising performances, there are still some concerns about their toxicity, which has been shown to depend mostly on the CNTs administration site, on their agglomeration state and on the presence of metal impurities in the material. The studies reported in this review indicate that CNTs seldom show toxicity toward brain tissues, probably thanks to the high purity and high dispersibility of the nanomaterials used. To date, the biggest impairment to the CNTs implementation in the therapy of CNS diseases is represented by the elimination of the catalyst metal nanoparticles, which requires the use of several procedures that hamper a cost-effective, large-scale production of high purity material.

Fullerenes have also shown good potential to find applications in the CNS, given their BBB crossing capabilities. The focus on their applications in the CNS however has been predominantly directed to exploit their intrinsic neuroprotective behavior, while other applications like drug delivery and imaging have been rarely investigated. Despite fullerenes can be synthesized in large quantities and at relatively low costs, their toxicity, which is more evident in water-soluble derivatives (i.e., the most appealing for the integration into a drug product) than in unfunctionalized fullerenes, represents a serious obstacle and currently limits their practical use in therapy.

Graphene oxide and derivatives can be successfully applied in the CNS for imaging applications by exploiting their intrinsic NIR fluorescence or the functionalization with MRI contrast agents. Moreover they can deliver therapeutic drugs and genetic material inside cerebral tumors. On the other hand, they are unable to cross the BBB unless appropriately functionalized or with the help of physical BBB opening methodologies. Although they tend to accumulate in lungs and they persist in the body for a longer time than other carbon nanomaterials, their low-toxicity profile, especially in the presence of extensive functionalization, makes them possible candidates for future implementations in therapy.

Nanodiamonds have been proposed very recently as suitable nanomaterials for biomedical applications. Indeed, studies of their application *in vivo* are still very limited compared to those available for other carbon nanomaterials. Nevertheless, NDs have already shown promising drug delivery capabilities inside the CNS, which allowed to obtain several improvements in the therapy of a cerebral tumor with doxorubicin. Moreover, NDs have not displayed any alarming toxicity. Although the research on possible diagnostic and therapeutic applications of NDs in the brain is in its infancy, the current results suggest that these nanomaterials may have an important role to play in the future brain medicine.

Carbon nanohorns and nanofibers demonstrate to have strong limitations for their direct application in the CNS: both of them do not show BBB crossing capabilities, and there are strong concerns regarding their toxicity and their strong accumulation in several organs. For these reasons the number of studies in the CNS conducted over the years is limited. We do not envisage therefore a future for the direct use of these nanomaterials in the therapy of CNS diseases.

Carbon dots are the latter discovered materials that have been examined for their possible implementation in the diagnosis and therapy of CNS diseases. For this reason, drug delivery or neuroprotection applications have not been evaluated *in vivo* yet. Nevertheless, biodistribution data indicate that carbon dots can cross the BBB very efficiently, and have a low retention in the body. Furthermore, there are only little evidences indicating their possible toxicity, which in any case seems to be very mild. Along with NDs, CDs therefore represent a very promising family of nanomaterials for future applications in brain diseases therapy and diagnosis. One current drawback is related to the fact that large-scale industrial production is still not technologically possible, even though the synthesis is relatively easy and cheap on a laboratory scale.

As highlighted in this review, research on carbon nanomaterials for brain-related applications is very active, and is taking full advantage of a combination of a broad spectrum of nanomaterials differing in their shape and properties and of a wealth of functional molecules that can provide them with additional tailored features. *In vivo* studies in the CNS represent a springboard for the implementation of carbon nanomaterials in the diagnosis and treatment of brain diseases. With this in mind, the studies herein presented offer an intriguing glimpse of what the continuous advancements in carbon nanomaterials technology will be able to provide in the near future. The number of different carbon nanostructures employed in brain research has increased dramatically in the last few years, yet there are some carbon nanomaterials that have not been investigated in this context already. Also, the success of NDs and CDs for biomedical applications indicates a higher potential for spherical carbon nanoparticles, both in terms of biocompatibility and efficiency in cell penetration. In this sense carbon nano-onions, which have been recently tested for possible biomedical use, have the potential to contribute positively to neurobiomedical applications. We are confident that in the near future the knowledge acquired on the different carbon nanomaterials features and on their toxicology, alongside with the progress made in maximizing their performances *in vivo*, will allow these nanomaterials to assume an important role in the diagnosis and treatment of brain diseases.

## Author contributions

SG and RT conceived the work. MB and MT performed the bibliographical search and drafted the manuscript. All authors contributed to the critical revision of the manuscript.

### Conflict of interest statement

The authors declare that the research was conducted in the absence of any commercial or financial relationships that could be construed as a potential conflict of interest.

## Abbreviations

ACh: acetylcholine
AD: Alzheimer's disease
FALS: familial amyotrophic lateral sclerosis
BBB: blood-brain barrier
CD: carbon dots
CED: convection-enhanced delivery
CNFs: carbon nanofibers
CNHs: carbon nanohorns
CNMs: carbon nanomaterials
CNOs: carbon nano-onions
CNS: central nervous system
CNTs: carbon nanotubes
CVD: chemical vapor deposition
DEX: dextran
DOX: doxorubicin
DTPA: diethylene triamine pentaacetic acid
EPI: epirubicin
FUS: focused ultrasounds
GFAP: glial fibrillary acidic protein
GI: gastrointestinal
GlcNF: glucosamine conjugate fullerenol
GO: graphene oxide
HIV: human immunodeficiency virus
*i.c.v*.: intracerebrovascular
*i.p*.: intraperitoneal
*i.v*.: intravenous
LFUS: low intensity focused ultrasound
MCAO: middle cerebral arteria occlusion
MPTP, 1-methyl-4-phenyl-1, 2, 3: 6-tetrahydropyridine
MRI: magnetic resonance imaging
MWCNTs: multi-wall carbon nanotubes
NDs: nanodiamonds
NK: natural killer
NO: nitric oxide
PCBM: phenyl-C61-butyric acid methyl ester
PD: Parkinson's disease
PEG: polyethylenglycol
PERF: perfenidone
PF-127: Pluronic F127
PVP: polyvinylpyrrolidone
QDs: quantum dots
RDX: cyclotrimethylenetrinitramine
ROS: reactive oxygen species
SCI: spinal cord injury
siRNA: small interfering RNA
SWCNHs: single-wall carbon nanohorns
SWCNTs: single-wall carbon nanotubes
TLR9: toll-like receptor 9
Tf: transferrin
TNT, 2, 4: 6-trinitrotoluene.
